# Method for redistributing ill-defined causes of death

**DOI:** 10.1080/00324728.2024.2332629

**Published:** 2024-04-26

**Authors:** Pavel Grigoriev, Florian Bonnet, Elsa Perdrix

**Affiliations:** 1 Federal Institute for Population Research; 2 French Institute for Demographic Studies; 3 Université Paris Dauphine; 4 PSL Research University

**Keywords:** mortality, ill-defined causes of death, method of redistribution

## Abstract

Analysis of causes of death is crucial for monitoring an epidemiological situation and for developing adequate policy responses. However, the comparability of cause-specific mortality data depends on the proportion of ill-defined deaths. To eliminate the bias resulting from the varying proportions of such causes over time and between populations, deaths from ill-defined causes need to be reassigned to other categories. We provide thorough documentation of and tools for the practical implementation of a regression-based method for redistributing ill-defined causes of death, as first proposed by Sully Ledermann in the 1950s. The method relies on subnational cause-specific mortality data to estimate unbiased death rates at both national and subnational levels. We refine Ledermann’s method by elaborating on its mathematical properties, making additional adjustments, and evaluating the performance of the approach through simulations. To illustrate the practical application of the method, we rely on French subnational cause-of-death data and provide the R code for performing all calculations.

## Introduction

Analysis of causes of death is crucial for monitoring an epidemiological situation and for developing adequate policy responses. However, such analysis is affected by the quality and comparability of cause-of-death data. Making cause-specific mortality data comparable across time and space involves addressing several methodological issues: for example, bridging different international classifications of diseases (ICD), dealing with statistical discontinuities in mortality trends due to coding practices, and redistributing deaths from ill-defined causes (Meslé and Vallin 2008; Pechholdová et al. 2017). Whereas a number of reasonable methods have been developed and implemented to mitigate the problems presented by discontinuities in cause-specific mortality trends (Meslé and Vallin 1996; Janssen and Kunst 2004; van der Stegen et al. 2014; Camarda 2019), there is no standard approach or well-established set of guidelines as regards dealing with ill-defined deaths.

Since there are various reasons as to why some deaths are given no specific diagnosis, the decision regarding an appropriate redistribution method depends on the specific context. There are two main approaches for dealing with the problem. The qualitative approach relies on detailed analysis of a few cases of ill-defined causes and/or on psychological autopsy (Cavanagh et al. 2003). The quantitative (data-driven) approach ensures a reasonable comparability of the observed number of deaths by cause between areas, population groups, and over time. The quantitative methods for reassigning deaths from ill-defined causes consist of two main approaches: (1) *proportional redistribution* (PR); and (2) redistribution using statistical models. Because of its simplicity and low requirements in terms of data availability, researchers and data analysts frequently rely on the PR method (Meslé et al. 1996; Pechholdová 2009; Grigoriev and Pechholdová 2017; World Health Organization 2022)*.* According to this method, ill-defined deaths are reassigned to other causes proportionally to their respective shares of the total number of deaths. The major drawback of the PR method is that it imposes the strong and unrealistic assumption that all causes have an equal chance of falling into the *ill-defined* category.

Redistribution methods using regression models have proven to be an efficient tool for harmonizing the large quantities of highly heterogeneous international data on causes of death. Most of these methods were developed as part of large-scale investigations conducted by World Health Organization and Global Burden of Disease experts for the treatment of incomplete data and, in particular, for redistributing so-called ‘garbage codes’ (Murray and Lopez 1996, 1997; Mathers et al. 2002; Lopez et al. 2006; Naghavi et al. 2010; Johnson et al. 2021). However, because of their complexity and the unavailability of computer code, these sophisticated techniques are hard to implement for small-scale studies involving only one or a few countries or just a few time points. For such cases, the regression-based method proposed by a French demographer, Sully Ledermann, appears to be a suitable solution (Ledermann 1955). This method relies on the correlation between ill-defined causes and deaths by cause at the subnational level. It is known only to a limited number of demographers specializing in the analysis of mortality by cause of death. Because the original paper was published in French and without an elaborated description of the applied redistribution method, its application in practice is rare (Paes and Gouveia 2010; Fihel and Meslé 2017).

In this paper, we provide thorough documentation of Ledermann’s method so that it can be easily implemented in practice. Building on the original publication, we introduce additional adjustments and describe the method in detail, highlighting its mathematical properties and discussing its assumptions and limitations. Another innovative aspect of this paper is its validation of Ledermann’s approach by way of a simulation test. To ensure that the results obtained can be reproduced, we rely on a freely accessible data set and provide R code for performing all calculations.

## Methods

Ledermann’s original method distributes deaths from causes classified as ‘ill-defined or unknown’ (hereafter simply *ill-defined*) among the other categories and allows unbiased death rates at the national level to be computed by using the information on causes of death at the local level. The method also allows the unbiased death rate by cause to be estimated in each local area. We describe next how both estimations are performed.

### Computing the unbiased death rate from cause c at the national level

For each geographical unit g(g⊂[[1,N]]) and each cause of death c(c⊂[[1,n]]), we note that:

*Z*_*cg*_ = the real but unknown proportion of deaths from cause *c* out of the total number of deaths in local area *g*.

To estimate this proportion, we need to define:

Θ_*cg*_ = the proportion of *Z*_*cg*_ classified as ill-defined.

Note that at the national level, we define $\Theta_{c}$ which is called the ‘dissimulation coefficient’. The only known parameters are:

X_*g*_ = the proportion of deaths from ill-defined causes in local area *g*.

Y_*cg*_ = the proportion of deaths from cause *c* in local area *g*.

**Figure 1:**
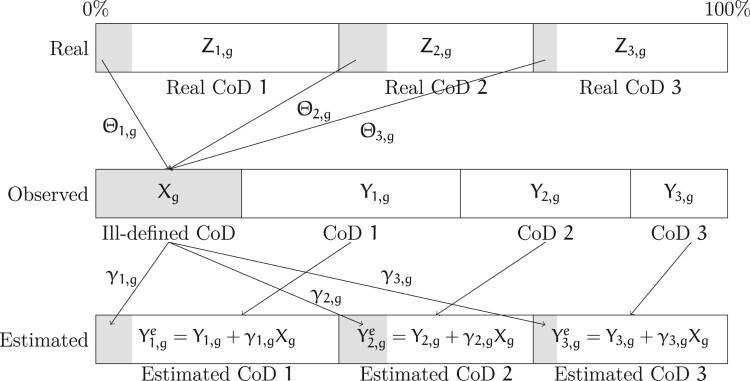
Ledermann’s method: Framework and notation *Note*: $\gamma_{c,g}$ are the estimated proportions of ill-defined $X_{g}$ attributable to cause of death c in local area *g*. Shaded areas show deaths originally classified as from ill-defined causes.

Figure 1 illustrates this framework. Let us assume that there are only four causes of death, of which three are well defined (CoD 1, CoD 2 and CoD 3) and one is ill-defined.

By definition, $X_{g}$ is equal to the sum of the proportions of total deaths that are ill-defined but are attributable to each cause of death c:

(1) $$\begin{array}{c} X_{g}=\overset{n}{\underset{c=1}{\sum }}Z_{\mathrm{cg}}\Theta_{\mathrm{cg}}. \end{array}$$

Further, $Y_{\mathrm{cg}}$ is equal to the real proportion of deaths from cause c: that is,$\;Z_{\mathrm{cg}}$ minus the proportion of deaths $Z_{\mathrm{cg}}$ that are classified as ill-defined:

(2) $$\begin{array}{c} Y_{\mathrm{cg}}=Z_{\mathrm{cg}}-Z_{\mathrm{cg}}\times \Theta_{\mathrm{cg}}=Z_{\mathrm{cg}}(1-\Theta_{\mathrm{cg}}). \end{array}$$

**Figure 2:**
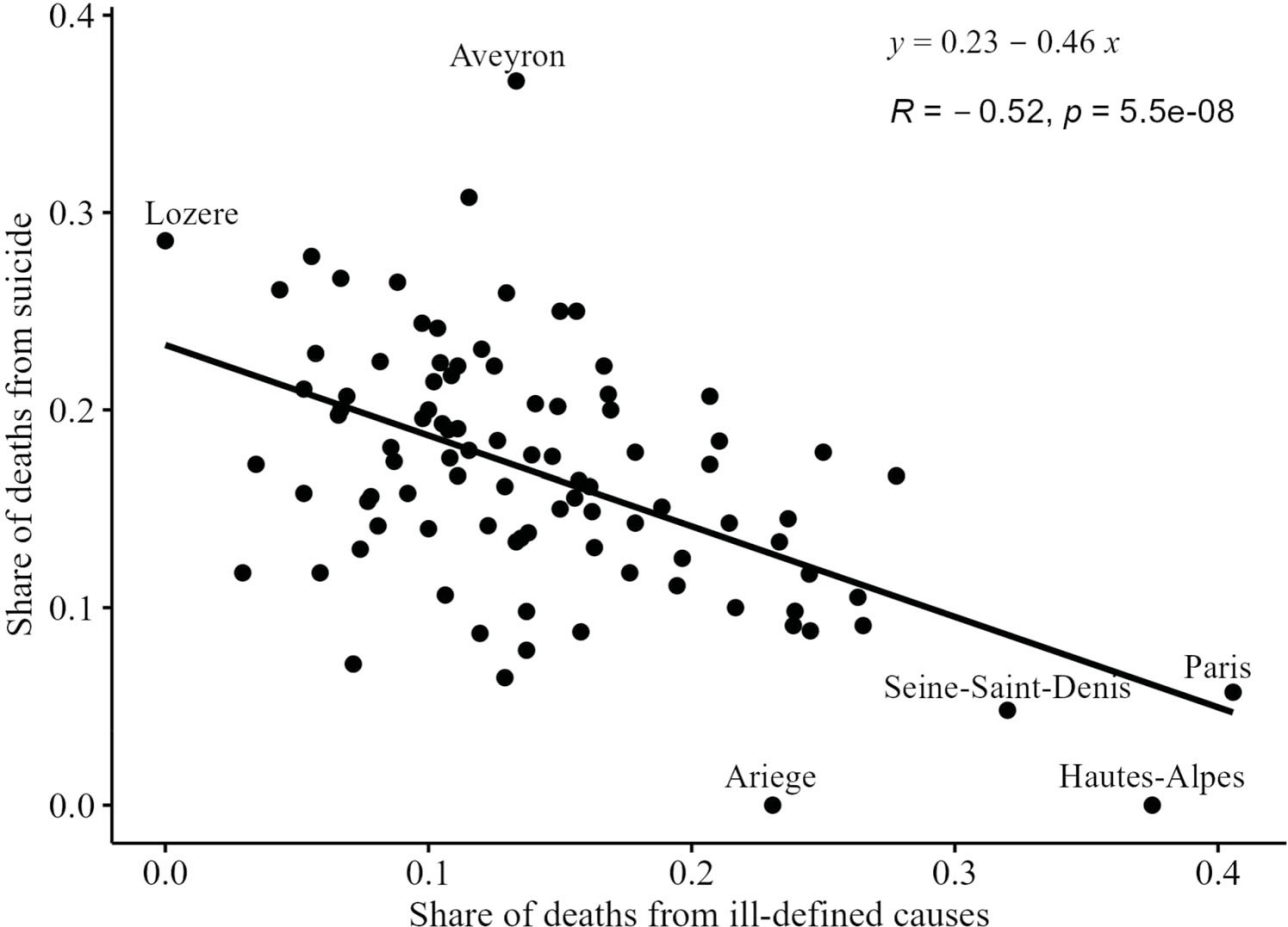
OLS regression in Ledermann’s method: An illustration for men in French departments with suicide as the cause of death *Note*: Each dot is a value at local level *g* (95 French departments), for men aged 35–44 in 2016. *Source*: Authors’ calculations based on official cause-of-death data from CépiDc (2020).

Ledermann (1955) estimated the real proportion of deaths from cause *c* in area g, $Z_{\mathrm{cg}}$, by determining the proportion of total deaths from cause c that would be observed at the national level if the proportion of ill-defined deaths were zero. To do this, we must use information on deaths by cause at the local level. This proportion is estimated using an ordinary least squares (OLS) regression with X as the explanatory variable and $Y_{c}$ as the dependent variable (see Figure 2 for an illustration using data for local areas in France).

For a given cause, c, we have:

(3) $$\begin{array}{c} Y_{\mathrm{cg}}=a_{c}-m_{c}X_{g}+\varepsilon_{\mathrm{cg}},\; \end{array}$$

with ε∼N(0,σ).

The regression leads to the following estimated parameters:

(4) $$\begin{array}{c} {\hat{Y}}_{\mathrm{cg}}={\hat{a}}_{c}-{\hat{m}}_{c}X_{g}. \end{array}$$

Thus, from the regression model (equation (4)) and based on assumptions we discuss later in this section, we obtain an estimation (${\hat{a}}_{c}$) of the true (but unobservable) proportion of deaths from cause c out of total deaths at the national level ($Z_{c}$). More interestingly, we obtain an estimation of $\hat{m_{c}}$. We show that $\hat{m_{c}}$ equalizes the average proportion of deaths from cause c that are hidden in ill-defined causes ($X_{g}$) over the average proportion of ill-defined deaths. In so doing, we obtain an estimation of the real number of deaths from cause c. Theoretically, $\sum_{c=1}^{n}{\hat{m}}_{c}=1$. Empirically, the sum can differ slightly from one, in particular at young ages. At these ages, the number of deaths could be low at subnational level and the shares more subject to randomness. In this case, we rescale all the $\hat{m_{c}}\;$ coefficients and obtain adjusted coefficients $\hat{{m^{\prime }}_{c}}$.

The estimated number of deaths from cause c is equal to the observed number of deaths plus $\hat{{m^{\prime }}_{c}}$ multiplied by the number of deaths that are classified as ill-defined. Lastly, we can compute the dissimulation coefficient for cause c ($\Theta_{c}$), which is the number of deaths from cause c hidden in ill-defined deaths over the real total number of deaths from cause c.

### Computing the unbiased death rate from cause c for each local area *g*

To estimate the unbiased death rate in each local area, we must use the information on the dissimulation coefficients $\Theta_{c}$ at the national level and the raw number of deaths from cause *c* at the local level. This allows an estimation of deaths classified as ill-defined cause to be inferred for cause of death c in each local area g. If we assume $\Theta_{\mathrm{cg}}$ to be close to the national average coefficient $\bar{\Theta_{c}}$, then since we measure $Y_{\mathrm{cg}}$, we can infer $Z_{\mathrm{cg}}$ (see equation (2)).

To allocate the national dissimulation coefficient to each local area, we define $p_{c}$, a weight of division equal to the dissimulation coefficient multiplied by the number of deaths from cause c in local area g, that is, $d_{\mathrm{cg}}$:

(5) $$\begin{array}{c} \;p_{c}=\Theta_{c}\times d_{\mathrm{cg}} \end{array}$$

Because of rounding, the number of deaths that are reallocated in this manner could differ slightly from the total number of ill-defined deaths. We therefore use the following iterative process:

(6) $$\begin{array}{c} {{\;p}^{\prime }}_{c}=p_{c}\left(1+\frac{\delta_{g}}{\sum_{k=1}^{n}p_{k}}\right),\; \end{array}$$

with $\delta_{g}$ being the number of deaths classified as ill-defined in local area g. Once stable weights of division are obtained, the proportion of deaths from cause *c* is obtained as follows.

First, we compute the total number of deaths from an ill-defined cause in local area g that are attributable to cause c: $r_{c}=\delta_{g}\times \gamma_{\mathrm{cg}}$ with $\gamma_{\mathrm{cg}}=\frac{\;p_{c}}{\sum_{k=1}^{n}p_{k}}$. Second, we add together the number of deaths from cause c in local area g and the estimated number of deaths from an ill-defined cause that are attributable to cause$c\;(r_{c})$: $D_{\mathrm{cg}}=d_{\mathrm{cg}}+r_{c}$. The estimated true proportion of deaths from cause c in local area g is therefore computed as $Y_{\mathrm{cg}}^{e}=\frac{D_{\mathrm{cg}}}{\sum_{k=1}^{n}D_{\mathrm{kg}}}$. Thus, $Y_{\mathrm{cg}}^{e}=Y_{\mathrm{cg}}+\gamma_{\mathrm{cg}}X_{g}$.

### Assumptions and limitations

In his original work, Ledermann did not discuss the assumptions and limitations of the model. Here, we highlight the explicit and implicit assumptions and discuss them to ensure that the method is used correctly.

*Assumption 1*: $X_{g}$ and $Z_{\mathrm{cg}}$ are independent, thus their covariance is zero. The residuals must follow a normal distribution, with no outliers. Since $Z_{c}$, the real proportion of deaths from cause *c*, is not observable, it is impossible to test this assumption formally. However, we can check the covariance of the estimated share of deaths from cause *c* $\left(Y_{\mathrm{cg}}^{e}\right)$ and X and generate scatter plots for each cause of death (similar to the one shown in Figure 2). Likewise, the distribution of the residuals can be visualized.

*Assumption 2*: The proportion of ill-defined deaths attributable to cause c over the proportion of deaths whose cause is classified as ill-defined in local area g is close to the national average, that is:

(7) $$\begin{array}{c} \forall g,\;\;\frac{Z_{\mathrm{cg}}\Theta_{\mathrm{cg}}}{X_{g}}\;\approx \frac{\bar{Z_{c}\Theta_{c}}}{\bar{X}}. \end{array}$$

In the supplementary material, section S1, we show that under Assumptions 1 and 2, $\hat{m_{c}}$ is equal to the average proportion of deaths from cause c hidden in deaths whose cause is classified as ill-defined over the average proportion of deaths whose cause is classified as ill-defined:

(8) $$\begin{array}{c} {\hat{m}}_{c}=\frac{\bar{Z_{c}\Theta_{c}}}{\bar{X}}\;. \end{array}$$

Moreover, $\;\hat{a_{c}}$ is equal to the average real proportion of deaths from cause c:

(9) $$\begin{array}{c} {\hat{a}}_{c}=\bar{Z_{c}}\;. \end{array}$$

Assumption 2 is highly credible because there is presumably a uniform process of registering deaths in all of the local areas considered. However, for historical or cross-national studies, Assumptions 1 and 2 cannot be verified.

It is hardly possible to test each assumption independently. However, one robustness check allows both assumptions to be assessed, meaning that a check can be carried out to see whether $\sum_{c=1}^{n}m_{c}$  =  1. Indeed,

(10) $$\begin{array}{rl} & \sum_{c=1}^{n}m_{c}=\sum_{c=1}^{n}\frac{\bar{Z_{c}\Theta_{c}}}{\bar{X}}\\ & \Leftrightarrow \sum_{c=1}^{n}m_{c}=\sum_{c=1}^{n}\frac{\bar{Z_{c}\Theta_{c}}}{\sum_{c=1}^{n}\bar{Z_{c}\Theta_{c}}}\\ & \Leftrightarrow \sum_{c=1}^{n}m_{c}=\sum_{c=1}^{n}\frac{\bar{Z_{c}\Theta_{c}}}{n(\bar{Z_{c}\Theta_{c})}}\\ & \begin{array}{c} \Leftrightarrow \sum_{c=1}^{n}m_{c}=\sum_{c=1}^{n}\frac{1}{n}=\frac{n}{n}=1. \end{array} \end{array}$$

Moreover, the coefficients $m_{c}$ are supposed to show minor variations between years. Note that this method relies on reaching a certain level of statistical power. Assumption 2 requires a low variance, that is, a certain degree of homogeneity between geographical areas. This can be achieved by applying the method to as many population subgroups as possible. However, such stratification eventually leads to a smaller number of observations, which in turn reduces the statistical power. Thus, a balance must be struck between statistical power and low variance to ensure the validity of Assumption 2. Moreover, as in all regressions, the higher the number of local areas, the more precise the estimates. Thus, the number of local areas and their size need to be balanced.

## Results

### Practical application: An example

To illustrate the practical implementation of Ledermann’s method, we use deaths by cause for 95 French local areas (departments), downloaded from the Centre d’épidémiologie sur les causes médicales de décès (CépiDc 2020). The data on population counts needed for computing deaths by age and sex and cause-specific deaths, respectively, originate from work carried out to produce French subnational life tables (Bonnet 2020). To produce our ‘Demo Dataset. xlsx’ (see supplementary material, section S2), we aggregate the raw data into 10-year age groups and 10 causes of death (see supplementary material, section S3, for details on the cause-of-death codification). Our task here is to redistribute Cause 10 (ill-defined) among Causes 1–9 in such a way that the adjusted mortality trends look plausible. To perform correct comparisons which account for the differences in population age structure, after applying each method for redistributing ill-defined deaths (PR or Ledermann’s), we compute the standardized death rate based on adjusted death counts and respective population exposures.

In Table 1, we provide an example that illustrates the process of redistributing ill-defined deaths (Cause 10) at the national level using French subnational data. We illustrate in charts the application of the method for males aged 35–44 (equivalent results for females appear in the supplementary material, section S4). We also consider the PR method as an alternative to Ledermann’s method.

**Table 1:** Death counts before and after redistributing deaths from ill-defined causes using the Ledermann and PR methods: Males aged 35–44, France, 2016

Cause	Reported deaths	Ledermann’s method			Proportional redistribution	
		OLS regression slope	Adjusted coefficient	Adjusted deaths	Coefficient	Adjusted deaths
Infectious diseases	81	0.003	0.003	84	0.016	96
Neoplasms	1,166	0	0	1,166	0.231	1,380
Diseases of the circulatory system	685	0.073	0.072	752	0.136	811
Diseases of the respiratory system	113	0.007	0.007	119	0.022	134
Diseases of the digestive system	396	0.073	0.071	462	0.079	469
Accidents	888	0.218	0.212	1,085	0.176	1,051
Suicides	956	0.459	0.447	1,370	0.190	1,132
Other external causes	141	0	0	141	0.028	167
Other causes of death	618	0.193	0.188	792	0.123	731
Ill-defined causes	927	–	–	–	–	–
Total	5,971	1.026	1.000	5,971	1.000	5,971

*Source*: Authors’ calculations based on official cause-of-death data from CépiDc (2020).

Among deaths of men in this age group, 15.5 per cent are reported as ill-defined. Except for neoplasms and for other external causes, the OLS regression returns negative slopes for all causes of death, implying that ill-defined deaths should be redistributed among those causes. The sum of the slopes, however, slightly exceeds the permitted value of 1.000. By rescaling those coefficients in a way that their sum yields 1.000, we ensure that ill-defined deaths are fully redistributed.

We apply the same procedure to each unique combination of year, sex, and age and for all selected causes of death, making sure that the total number of deaths in each stratum before and after the redistribution remains the same. The sequence of the computational steps is presented in the R script ‘Demo R Code.R’, which is accompanied by extensive comments (see supplementary material, S2). Briefly, we calculate the proportions of the total number of deaths that are from each cause of death using the function ‘ShareCD’. We then estimate the transition coefficients (function ‘CoeffLed’). This can be done with or without applying the smoothing procedure across ages. The smoothing procedure is carried out with local regressions, and users are able to alter the smoothing parameter. Finally, we apply the obtained coefficients to estimate ‘adjusted death counts at the department level (function ‘CoeffLedRedist’).

**Figure 3:**
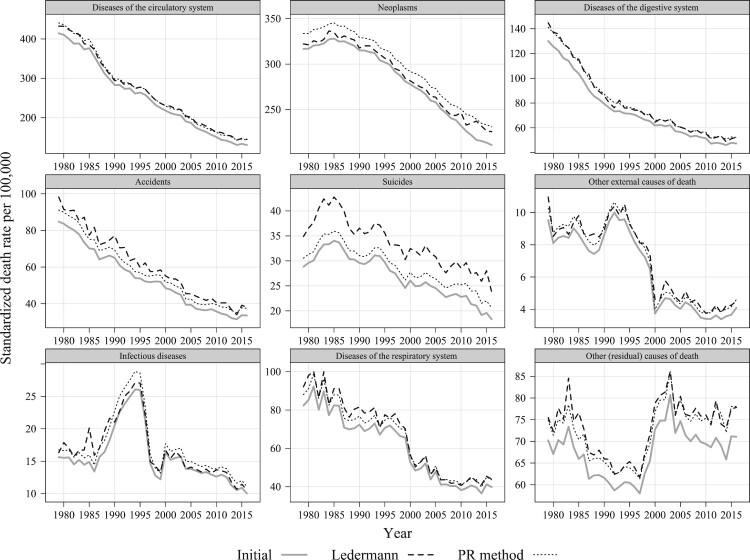
Mortality trends by selected causes of death before and after redistributing deaths from ill-defined causes using the Ledermann and PR methods: Males aged 35–44, France, 1979–2016 *Source*: As for Figure 2.

Figure 3 depicts the initial mortality trends (based on the reported data) and adjusted trends for France by our nine cause-of-death groups. The application of Ledermann’s method results in a substantial upward correction in mortality from suicides, which is consistent with the fact that this cause of death reveals the strongest statistical association with ill-defined causes (Table 1). By contrast, using the simple PR method produces a rather modest correction. However, that approach reassigns considerably more deaths to neoplasms, one of the leading causes of death and one of the most reliable in terms of the quality of registration. It is unrealistic to assume that so many cancer deaths are misclassified as ill-defined. As far as other causes of death are concerned, the application of both methods produces very similar results.

Ledermann’s method not only allows the national-level data to be adjusted by using subnational data but also allows the subnational data themselves to be corrected. Both adjustments are equally important in practical applications. To demonstrate the performance of the method at subnational level, we select several French departments. These departments include the six outliers highlighted in Figure 2 (Aveyron, Ariège, Hautes-Alpes, Lozère, Paris, Seine-Saint-Denis) and two other departments with distinct features: (1) Alpes-Maritimes—an urban department in the south with a high share of deaths from ill-defined causes, distinctive trends in suicide mortality, and low all-cause mortality; and (2) Pas-de-Calais—a poor urban department which for decades has experienced the highest mortality in France. Paris is the largest urban department and reports a high share of ill-defined causes. Seine-Saint-Denis, a large Paris suburb, represents the poorest French department and displays very high mortality. By contrast, Ariège is a rural department, with a relatively high share of deaths from ill-defined causes and low suicide mortality. Aveyron is another small, predominantly rural department with a low share of deaths from ill-defined causes but high suicide mortality.

**Figure 4:**
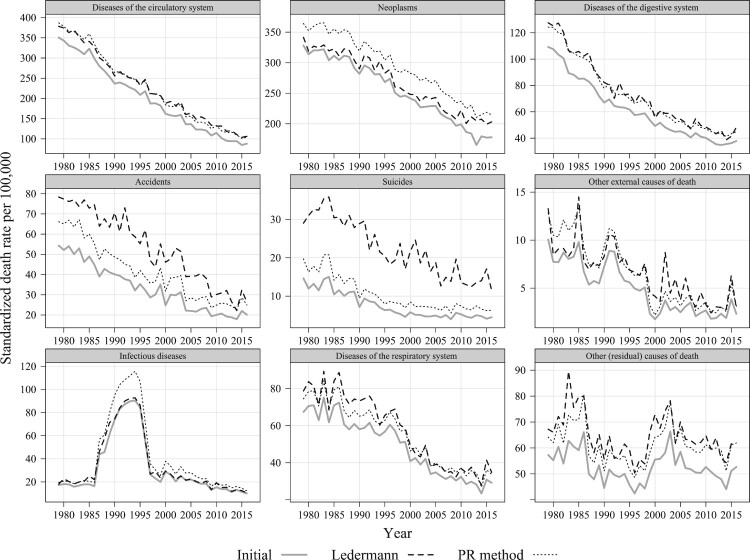
Mortality trends by selected causes of death before and after redistributing deaths from ill-defined causes using the Ledermann and PR methods: Males aged 35–44, Paris, 1979–2016 *Source*: As for Figure 2.

The adjustments to men’s mortality trends as applied to the Paris department are shown in Figure 4. The results for women in Paris and results for the other selected departments appear in the supplementary material, S4.

As for the national mortality trends (Figure 3), the application of Ledermann’s method to the Paris department produces plausible results. As expected, compared with the PR method, Ledermann’s approach assigns fewer deaths to neoplasms and more deaths to accidents and, in particular, suicides.

**Figure 5:**
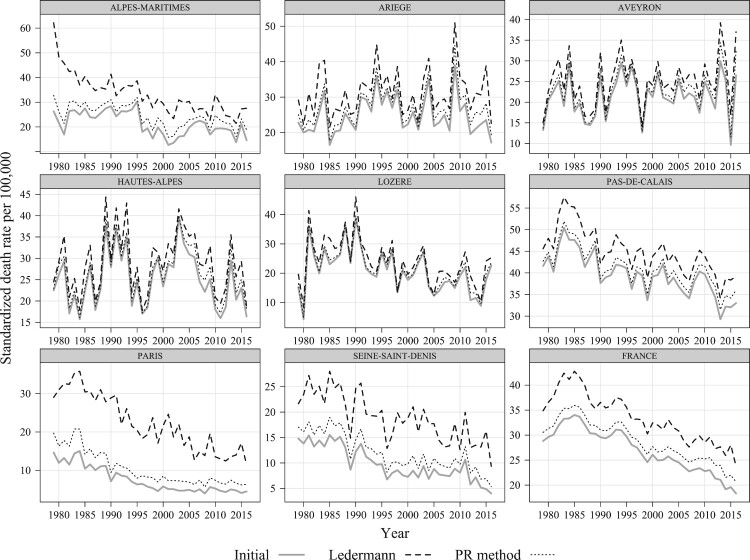
Mortality from suicide in selected departments before and after redistributing deaths from ill-defined causes using the Ledermann and PR methods: Males aged 35–44, France, 1979–2016 *Source*: As for Figure 2.

The degree to which the latter is corrected varies by department (Figure 5). It appears that the departments with high proportions of deaths from ill-defined causes and low reported suicide mortality (Alpes-Maritimes, Seine-Saint-Denis, and Paris) are affected most by the adjustments. Strong subnational differences in the level of suicide misclassification are likely attributable to subnational differences in coding, suicide definitions, confidentiality regulations, and religious and cultural context (Andriessen 2006; Richaud-Eyraud et al. 2018). At the same time, the departments with relatively small shares of deaths from ill-defined causes (e.g. Lozère) are less sensitive to the choice of redistribution method. On the whole, Ledermann’s method produces plausible results for both males and females in all cases. It is also clear that when the number of deaths is relatively small, Ledermann’s method generates more fluctuating trends compared with the PR method (supplementary material, S4).

### Model validation using simulated data

To validate the better performance of Ledermann’s method against the PR method, we run a simulation test (see also the R script ‘Simulation test.R’). First, we simulate a data set, called ‘real data’ (Data1 in the R code), which contains the real number of deaths for each of 95 departments for three causes of death. The shares of deaths from each cause are noted as $Z_{1},\;\;Z_{2},\;\;Z_{3}$, respectively. They follow a multivariate normal distribution that is mutually independent. We then simulate the θ coefficients conforming to Assumptions 1 and 2. These variables follow a normal distribution with a low variance. They are simulated in such a way that the covariance between the share of ill-defined deaths (*X*) and $Z_{i\;}$ is equal to zero. Lastly, we calculate the share of ill-defined causes of death *X*, where $X=\theta_{1}Z_{1}+\theta_{2}Z_{2}+\theta_{3}Z_{3}$.

**Figure 6:**
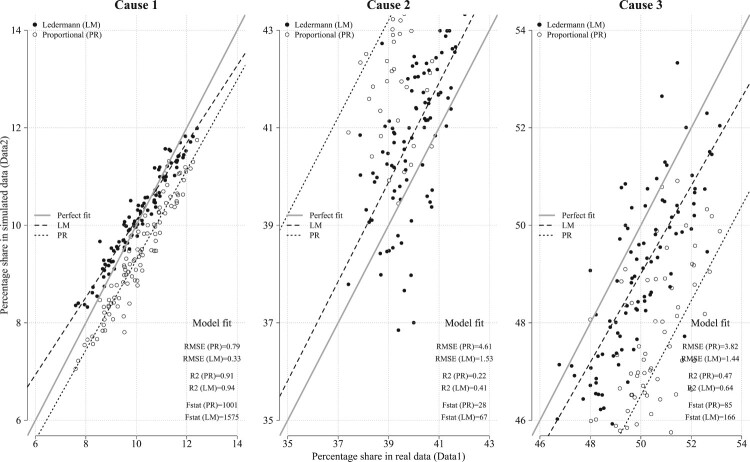
Performance of the Ledermann method against the simple PR method *Source*: Authors’ calculations based on simulated data for 95 geographical units and three causes of death.

Based on these simulated data, we create a new data set, ‘simulated data’ (Data2 in the R code), which contains the observed values in the presence of ill-defined deaths (i.e. a share of deaths $Y_{1},\;\;Y_{2},\;\;Y_{3}$ and a share of ill-defined causes of death, *X*). We then apply the PR method and Ledermann’s method to the ‘simulated data’ and assess whether the estimates obtained are close to the ‘real data’. The results are shown in Figure 6.

Each panel of the figure focuses on a particular cause of death. For each geographical unit, the graph shows the share of deaths from cause *c* in ‘real data’ (x-axis) and the share of deaths from cause *c* estimated through Ledermann’s method and by the PR method (y-axis). The grey solid line represents the perfect fit (i.e. where the estimated share of death is exactly equal to the share of death in ‘real data’). The results suggest that Ledermann’s method outperforms the PR method. Moreover, the former produces a better model fit than the latter, as indicated by the root mean square error (RMSE), R^2^, and F statistic.

**Figure 7:**
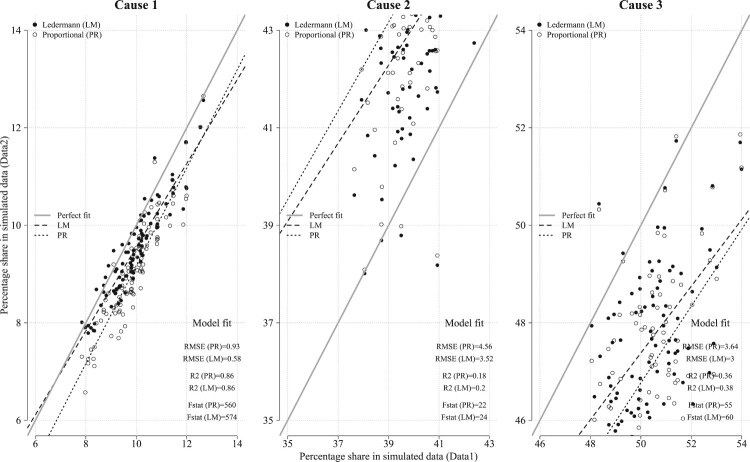
Performance of the Ledermann method against the simple PR method under the violation of Assumption 1 *Source*: As for Figure 6.

However, the efficiency of Ledermann’s method can no longer be guaranteed where its underlying assumptions are violated. Figure 7 shows the results of the application of Ledermann’s method in the presence of a high variance of the dissimulation coefficient theta, which results in the violation of Assumption 1. In this case, Ledermann’s method still performs reasonably well as far as Cause 1 is concerned. However, it produces unsatisfactory results for Causes 2 and 3. The same observations apply for the PR method. That is why it is important to verify and ensure that the sum of *m* is equal to one. Overall, the simulation test suggests that under the assumptions discussed earlier, the application of Ledermann’s method tends to produce results that are closer to the ‘true but unobservable’ data.

This simulation exercise also allows us to test how sensitive the method is to variation in the number of subnational areas (see supplementary material, section S5A). It shows that regardless of the number of areas, the statistical properties of Ledermann’s method are always better and give a prediction closer to the reality than the PR method does. The only instance where this is not the case is when the number of subnational areas is very small, because the regression models appear to lack statistical power when the sample size is too small.

We also take advantage of this simulation to test the sensitivity of the two methods to the share of ill-defined causes. We show that the higher the share of ill-defined causes, the better the relative performance of Ledermann’s method (see supplementary material, S5B).

## Conclusion

In this paper we present the first comprehensive documentation of Ledermann’s method, a simple and elegant approach for redistributing ill-defined causes of death. The example we use to demonstrate its application is reproducible, as we rely on open-access mortality data and provide a computer code for all calculations. All these factors should facilitate a wider usage of the method in practice. Ledermann’s method can be applied not only for the purposes of adjusting national cause-specific mortality data using subnational data but also for correcting subnational data themselves. This feature of Ledermann’s method is unique in that it allows for coherence between national and subnational mortality estimates. Furthermore, use of the method in practice is not limited to the redistribution of ill-defined causes. The method can easily be adapted for reassigning garbage codes or any cause(s) within any desired cause-of-death group. The availability of the code will facilitate the extension of this method by researchers. Several potential extensions (improvements) are possible: for example, the integration of weighting into the method to take account of differences in population size between local areas.

## Supplementary Material

Supplemental Material
