# Supplementary material for: Method for redistributing ill-defined causes of death
Source: Popul Stud (Camb). 2024 Apr 26;79(1):187–97. doi: 10.1080/00324728.2024.2332629 (PMC11956782; doi:10.1080/00324728.2024.2332629)

Proof of $\boldsymbol{a_c = \overline{Z_c}}$

By definition, we have the sum of the ill-defined cause of death in local area g which is the sum of the share of ill-defined cause death attributable at each cause of death c:

$$X_g = \sum_{c=1}^{n} Z_{cg}\Theta_{cg} \tag{1}$$

Moreover, we have the share of death of cause c observed in local area g which is the real share of death minus the share of unknown cause of death which would be attributable to this cause of death c:

$$Y_{cg} = Z_{cg}\left(1 - \Theta_{cg}\right) \tag{2}$$

We have:

$$\forall\, c \in [1, n], \forall\, g \in [1, N]\text{: } Y_{cg} = a_c - m_c X_g + \varepsilon_{cg} \tag{3}$$

With $\varepsilon \sim \mathcal{N}(0, \sigma)$ the residual, following a normal distribution.

This equation is estimated using an ordinary least square. Thus, we estimate the regression line, for each cause of death c, in the following regression:

$$\hat{Y}_{cg} = \hat{\mathrm{a}}_{\mathrm{c}} - \widehat{\mathrm{m}}_c X_g \tag{4}$$

To avoid a too heavy notation, in what follow, the hat is not mentioned while we systematically refers to the estimated values of Y, a and m.

By summing the N Equations 4, we obtain:

$$\sum_{g=1}^{N} Y_{cg} = N a_c - m_c \sum_{g=1}^{N} X_g \tag{5}$$

We also have, multiplying Equation 4 by X_g and thereafter summing the N equations:

$$X_g Y_{cg} = a_c X_g - m_c X_g^2 \Leftrightarrow \sum_{g=1}^{N} X_g Y_{cg} = a_c \sum_{g=1}^{N} X_g - m_c \sum_{g=1}^{N} X_g^2 \tag{6}$$

Combining Equations 5 and 6, we have:

$$\begin{cases} \sum_{g=1}^{N} Y_{cg} = Na_c - m_c \sum_{g=1}^{N} X_g \\ \sum_{g=1}^{N} X_g Y_{cg} = a_c \sum_{g=1}^{N} (X_g) - m_c \sum_{g=1}^{N} (X_g^2) \end{cases} \tag{7}$$

$$\begin{cases} a_c = \frac{1}{N} \sum_{g=1}^{N} Y_{cg} + \frac{1}{N} m_c \sum_{g=1}^{N} X_g \\ \sum_{g=1}^{N} X_g Y_{cg} = (\frac{1}{N} \sum_{g=1}^{N} Y_{cg} + \frac{1}{N} m_c \sum_{g=1}^{N} X_g) \sum_{g=1}^{N} X_g - m_c \sum_{g=1}^{N} X_g^2 \end{cases} \tag{8}$$

$$\Leftrightarrow \sum_{g=1}^{N} X_g Y_{cg} = \frac{1}{N} \sum_{g=1}^{N} Y_{cg} \sum_{g=1}^{N} X_g + \frac{m_c}{N} \sum_{g=1}^{N} X_g \sum_{g=1}^{N} X_g - m_c \sum_{g=1}^{N} X_g^2 \tag{9}$$

$$\Leftrightarrow \sum_{g=1}^{N} X_g Y_{cg} = \frac{1}{N} \sum_{g=1}^{N} Y_{cg} \sum_{g=1}^{N} X_g + m_c \left(\frac{1}{N} \left(\sum_{g=1}^{N} X_g \right)^2 - \sum_{g=1}^{N} X_g^2 \right) \tag{10}$$

$$m_c = \frac{\sum_{g=1}^{N} X_g Y_{cg} - \left(\frac{1}{N} \sum_{g=1}^{N} Y_{cg} \sum_{g=1}^{N} X_g \right)}{\frac{1}{N} \left(\sum_{g=1}^{N} X_g \right)^2 - \sum_{g=1}^{N} X_g^2} \tag{11}$$

$$m_c = \frac{- \sum_{g=1}^{N} X_g Y_{cg} + \bar{X} \sum_{g=1}^{N} Y_{cg}}{-N\, \bar{X}^2 + \sum_{g=1}^{N} X_g^2} \tag{12}$$

We note A the numerator of m_c. Thus, $A = - \sum_{g=1}^{N} X_g Y_{cg} + \overline{X} \sum_{g=1}^{N} Y_{cg}$.

Assumption 1: X and Z are independent (their covariance equal zero). Under this assumption, we have (proof of Equation 13 below):

$$A \Leftrightarrow \sum_{g=1}^{N} (X_g^2 - \bar{X} X_g) (\frac{Z_{cg} \Theta_{cg}}{X_g}) \tag{13}$$

Assumption 2: The share of unknown death attributable to cause c over the total unknown death in local area g is very close to the average, ie: $\forall g, \frac{Z_{cg}\Theta_{cg}}{X_g} \approx \frac{\overline{Z_c \Theta_c}}{\overline{X}}$

Under this assumption, we have:

$$\Leftrightarrow A = \sum_{g=1}^{N} (X_g^2 - \bar{X} X_g) (\frac{\overline{Z_c \Theta_c}}{\bar{X}}) \tag{14}$$

As a consequence, we can rewrite Equation 12 as following:

$$m_c = \frac{\sum_{g=1}^{N} (X_g^2 - \bar{X} X_g) (\frac{\overline{Z_c \Theta_c}}{\bar{X}})}{-N\, \bar{X}^2 + \sum_{g=1}^{N} X_g^2} \tag{15}$$

$$\Leftrightarrow m_c = \frac{\sum_{g=1}^{N}(X_g^2 - \bar{X}X_g)(\frac{\overline{Z_c\Theta_c}}{\bar{X}})}{\sum_{g=1}^{N}(X_g^2 - \bar{X}X_g)} \quad (16)$$

Thus, under assumptions 1 and 2:

$$m_c = \frac{\overline{Z_c\Theta_c}}{\bar{X}} \quad (17)$$

Once the parameter m_c determined, it is possible to deduct the value of parameter a_c. Since the deduction is from Equation 17, the following holds only under assumption 1 and 2.

$$\forall g \in [\![1, N]\!]: a_c = Y_{cg} + m_c X_g \Leftrightarrow a_c = Y_{cg} + \frac{\overline{Z_c\Theta_c}}{\bar{X}} X_g \quad (18)$$

Thus, we have a system of N equations. By summing we obtain:

$$N a_c = \sum_{g=1}^{N} Y_{cg} + \sum_{g=1}^{N} \frac{\overline{Z_c\Theta_c}}{\bar{X}} X_g \Leftrightarrow a_c = \bar{Y_c} + \frac{\overline{Z_c\Theta_c}}{\bar{X}} \frac{\sum_{g=1}^{N} X_g}{N} \quad (19)$$

$$\Leftrightarrow a_c = \bar{Y_c} + \overline{Z_c\Theta_c} \quad (20)$$

Since $\bar{Y_c} = \sum_{g=1}^{N} \frac{Z_{cg}(1-\Theta_{cg})}{N} = \overline{Z_c} - \overline{Z_c\Theta_c}$, we have:

$$a_c = \overline{Z_c} - \overline{Z_c\Theta_c} + \overline{Z_c\Theta_c} \;\Leftrightarrow\; a_c = \overline{Z_c} \quad (21)$$

What needed to be shown.

Proof of Equation 13

First, we replace in A, Y_{cg} using Equation (2):

$$A = -\sum_{g=1}^{N} X_g \left(Z_{cg}(1 - \Theta_{cg})\right) + \bar{X} \sum_{g=1}^{N} Z_{cg}(1 - \Theta_{cg}) \quad (22)$$

Then, we develop:

$$A \Leftrightarrow -\sum_{g=1}^{N} X_g Z_{cg} + \sum_{g=1}^{N} X_g Z_{cg}\Theta_{cg} + \bar{X}\sum_{g=1}^{N} Z_{cg} - \bar{X}\sum_{g=1}^{N} Z_{cg}\Theta_{cg} \quad (23)$$

We add $\overline{Z_c}\sum_{g=1}^{N} X_g - \overline{Z_c}\sum_{g=1}^{N} X_g$ to the previous equation:

$$A \Leftrightarrow -\sum_{g=1}^{N} X_g Z_{cg} + \sum_{g=1}^{N} X_g Z_{cg}\Theta_{cg} + \bar{X}\sum_{g=1}^{N} Z_{cg} - \bar{X}\sum_{g=1}^{N} Z_{cg}\Theta_{cg} + \overline{Z_c}\sum_{g=1}^{N} X_g - \overline{Z_c}\sum_{g=1}^{N} X_g \quad (24)$$

We re-arrange the order of the parameters in the sum:

$$A \Leftrightarrow -\sum_{g=1}^{N} X_g Z_{cg} + \overline{Z_c}\sum_{g=1}^{N} X_g + \bar{X}\sum_{g=1}^{N} Z_{cg} - \overline{Z_c}\sum_{g=1}^{N} X_g + \sum_{g=1}^{N} X_g Z_{cg}\Theta_{cg} - \bar{X}\sum_{g=1}^{N} Z_{cg}\Theta_{cg}$$

$$A \Leftrightarrow -\sum_{g=1}^{N} (X_g Z_{cg} - \overline{Z_c}X_g - \bar{X}Z_c + \overline{Z_c}X_g) + \sum_{g=1}^{N} X_g Z_{cg}\Theta_{cg} - \bar{X}\sum_{g=1}^{N} Z_{cg}\Theta_{cg}$$

$$A \Leftrightarrow -\sum_{g=1}^{N} ((X_g - \bar{X})(Z_{cg} - \overline{Z_c})) + \sum_{g=1}^{N} X_g Z_{cg}\Theta_{cg} - \bar{X}\sum_{g=1}^{N} Z_{cg}\Theta_{cg} \qquad (25)$$

Since we assume X and Z to be independent, $\sum_{g=1}^{N}((X_g - \bar{X})(Z_{cg} - \overline{Z_c})) = 0$. Thus,

$$A \Leftrightarrow \sum_{g=1}^{N} X_g Z_{cg}\Theta_{cg} - \bar{X}\sum_{g=1}^{N} Z_{cg}\Theta_{cg}$$

$$A \Leftrightarrow \sum_{g=1}^{N} \frac{X_g^2 Z_{cg}\Theta_{cg}}{X_g} - \bar{X}\sum_{g=1}^{N} \frac{Z_{cg}\Theta_{cg}X_g}{X_g}$$

$$A \Leftrightarrow \sum_{g=1}^{N} (X_g^2 - \bar{X}X_g)(\frac{Z_{cg}\Theta_{cg}}{X_g}) \qquad (13)$$

Online Supplementary Appendix S2

Brief description of the input data and R codes* used to demonstrate the application of the Ledermann's method

File	Description
Data	
Demo Dataset.xlsx	Death counts by region (95 French departments), sex, cause of death, and age; 1979 and 2016
Population.xlsx	Population at risk by region (95 French departments), sex, and age; 1979 and 2016
R codes	
Demo R Code.R	Example of the application of Ledermann's method using real data (*'Demo Dataset.xlsx' and 'Population.xlsx'*)
Simulation test.R	Validation of the Ledermann's model: a general simulation test
Simulation test_N.R	Validation of the Ledermann's model: testing performance depending on the number of regions
Simulation test_share of illdefined.R	Validation of the Ledermann's model: testing performance depending on the share of ill-defined causes

***the input data and the R codes can be downloaded from**

https://figshare.com/s/8b810e093fc32bcbe890

DOI: 10.6084/m9.figshare.19762408

Online Supplementary Appendix S3

List of causes of death with the correspondence to the items of the ICD–9 and the ICD–10

	Name	ICD–9	ICD–10
1	Infectious diseases	001–139	A00–B99
2	Neoplasms	140–239	C00–D48
3	Diseases of the circulatory system	390–459	I00–I52, I60–I99, G45
4	Diseases of the respiratory system	460–519	J00–J22, J30–J98, U04
5	Diseases of the digestive system	520–579	K00–K93
6	Accidents	E800–E949	V01–V99, W00–W99, X00–X59, Y40–Y98
7	Suicides	E950–E959	X60–X84
8	Other external causes of death	E960–E999	X85–Y09, Y35, Y36, Y10–Y34
9	Other (residual) causes of death	*REST*	*REST*
10	Ill-defined and unspecified causes of death	780–799	R00–R54, R56–R99

Online Supplementary Appendix S4

Mortality trends by selected causes of death before and after redistributing ill-defined causes of deaths; selected French departments, 1979–2016

Alpes-Maritimes

MALES

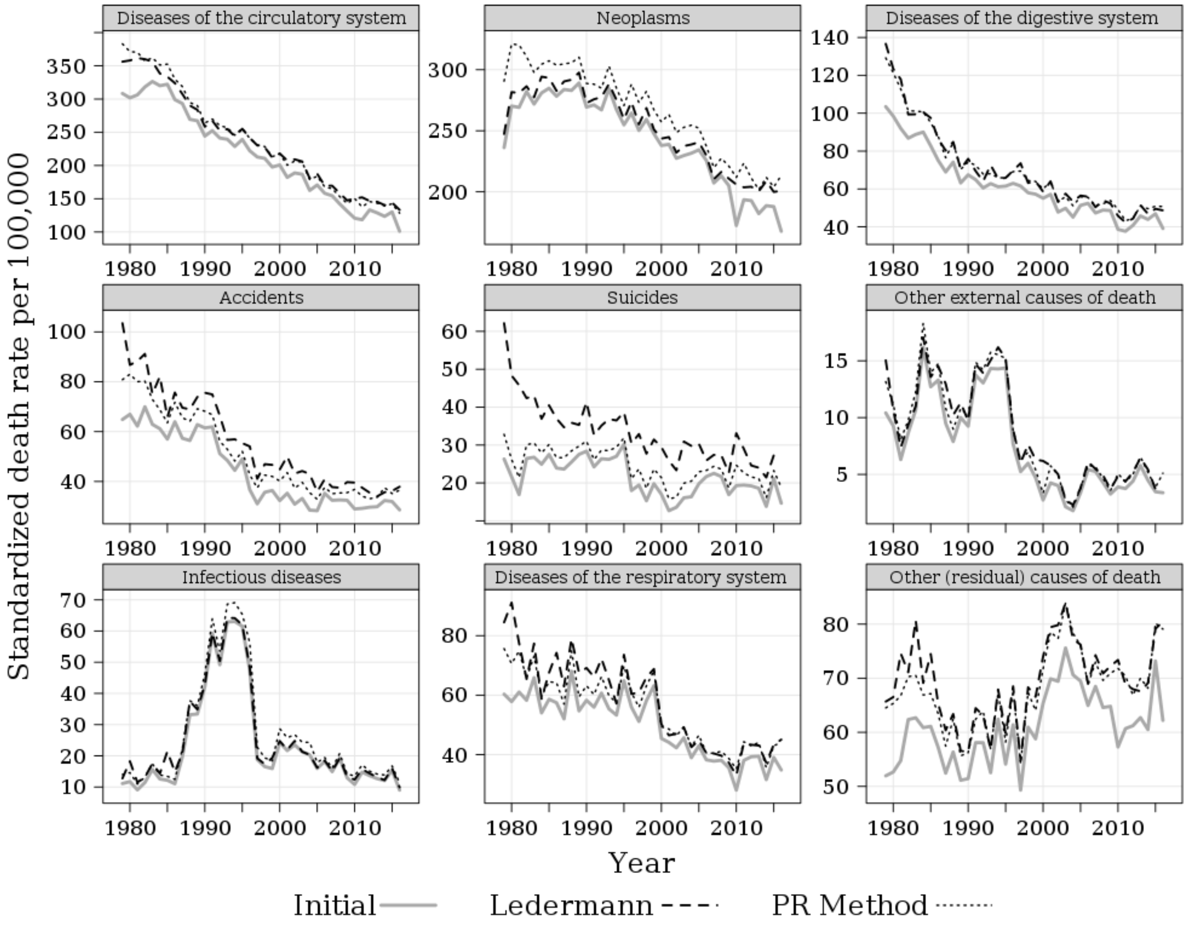

FEMALES

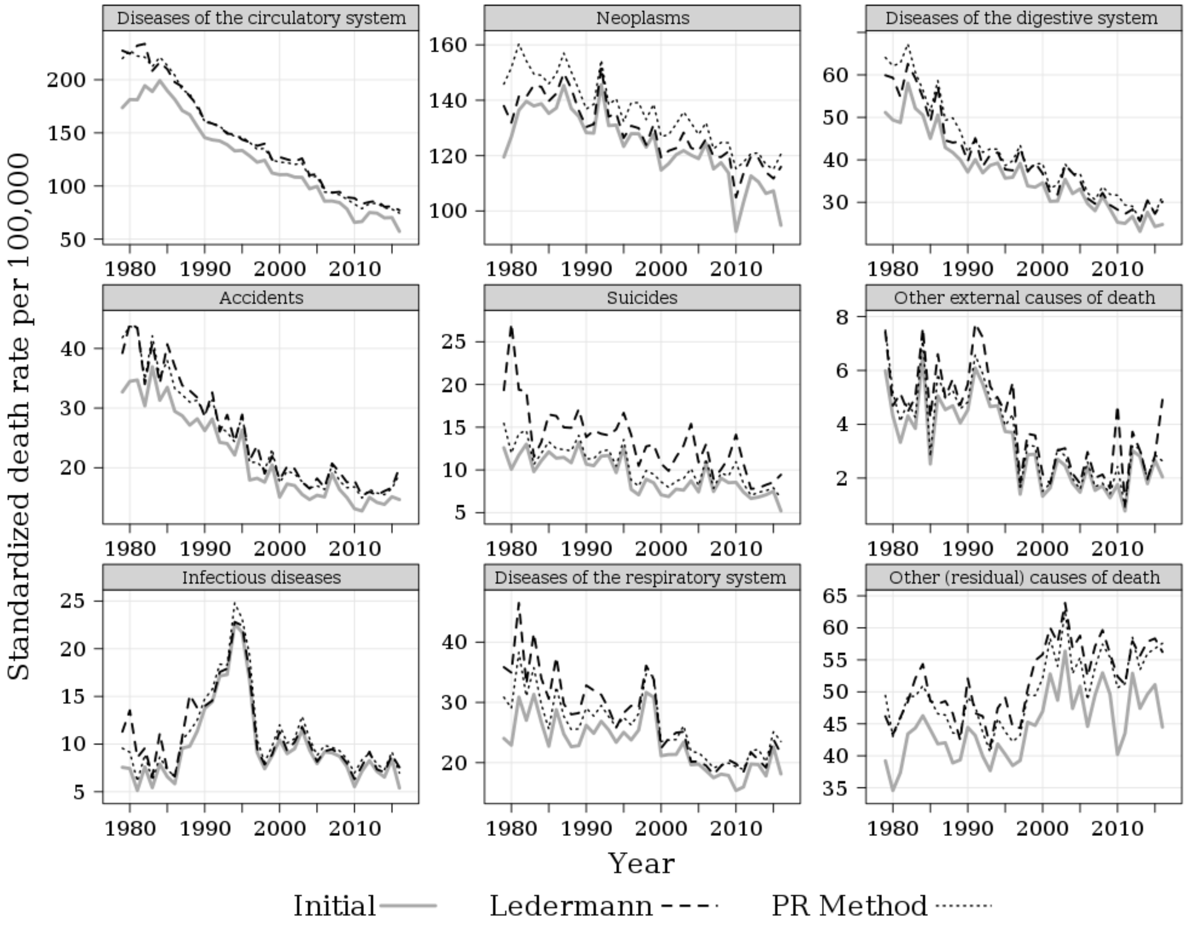

Diseases of the circulatory system
Neoplasms
Diseases of the digestive system
Accidents
Suicides
Other external causes of death
Infectious diseases
Diseases of the respiratory system
Other (residual) causes of death
Standardized death rate per 100,000
Year
Initial
Ledermann
PR Method

Aveyron

MALES

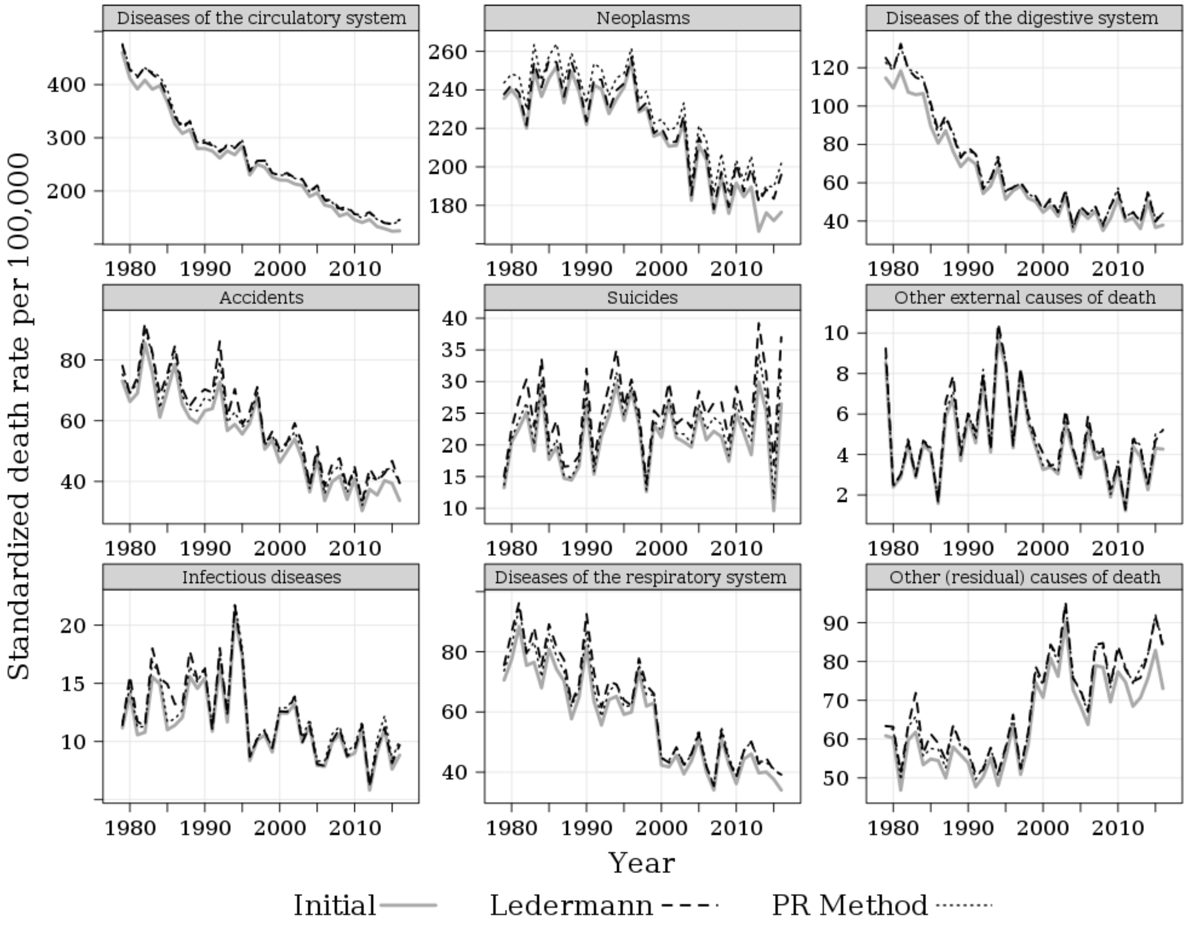

FEMALES

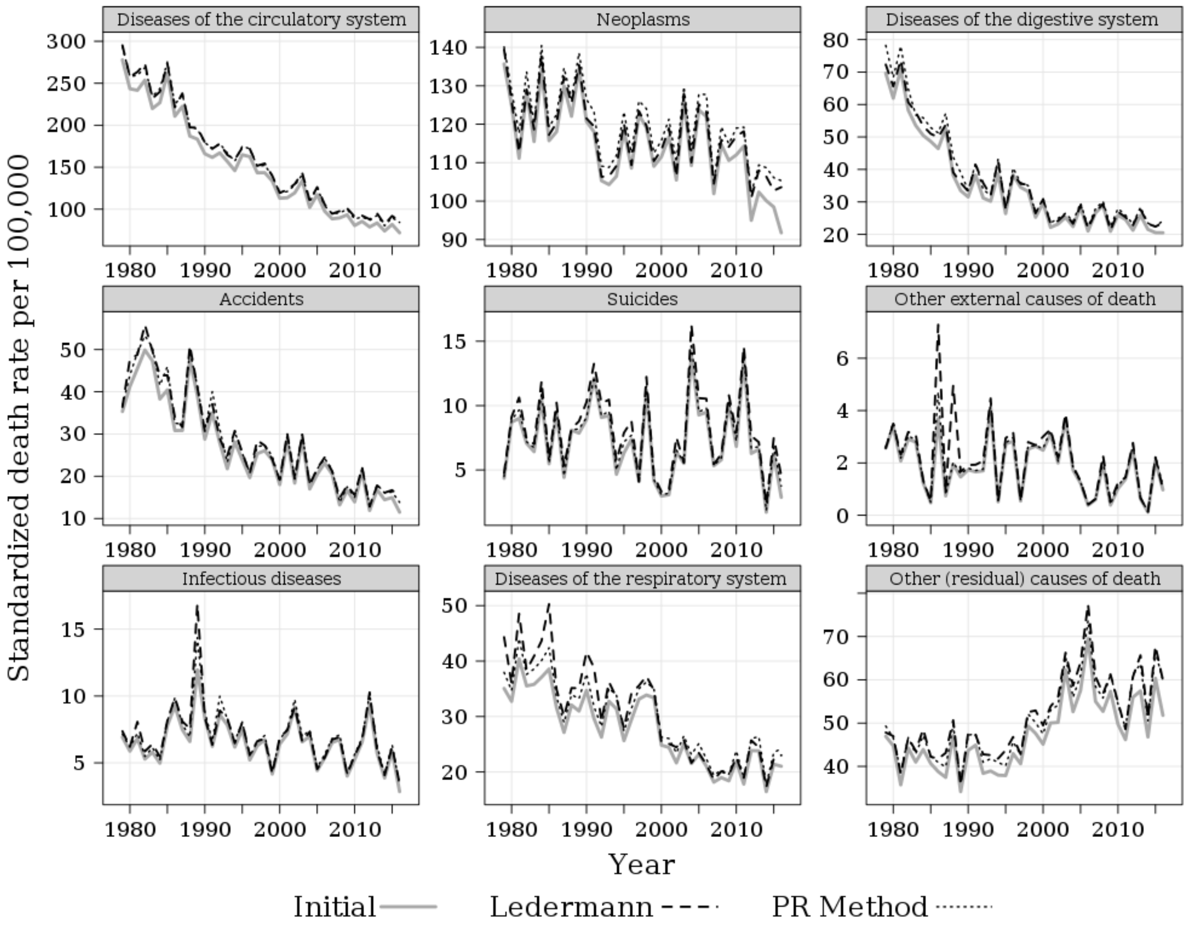

Diseases of the circulatory system
Neoplasms
Diseases of the digestive system
Accidents
Suicides
Other external causes of death
Infectious diseases
Diseases of the respiratory system
Other (residual) causes of death
Standardized death rate per 100,000
Year
Initial
Ledermann
PR Method

Ariège

MALES

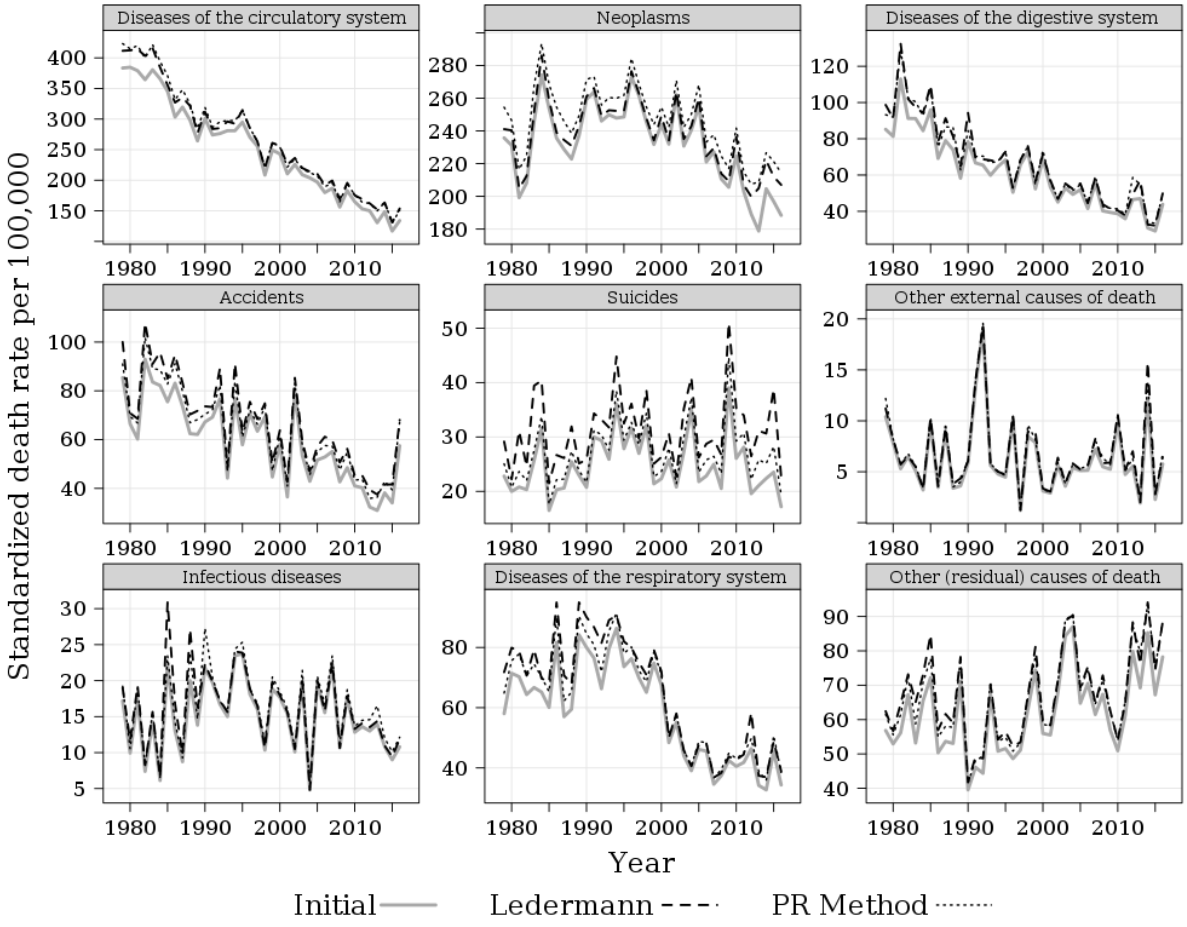

FEMALES

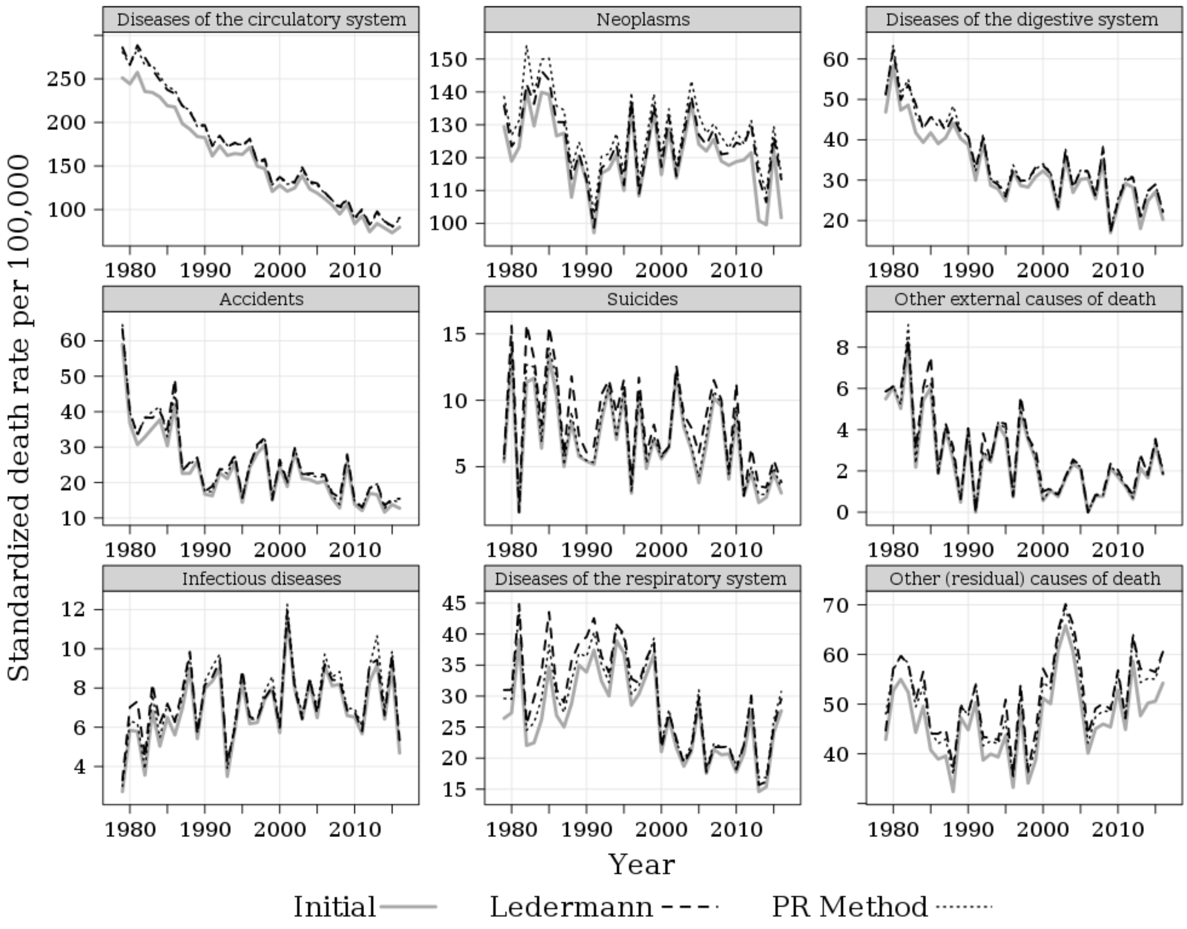

Diseases of the circulatory system
Neoplasms
Diseases of the digestive system
Accidents
Suicides
Other external causes of death
Infectious diseases
Diseases of the respiratory system
Other (residual) causes of death
Standardized death rate per 100,000
Year
Initial
Ledermann
PR Method

Hautes-Alpes

MALES

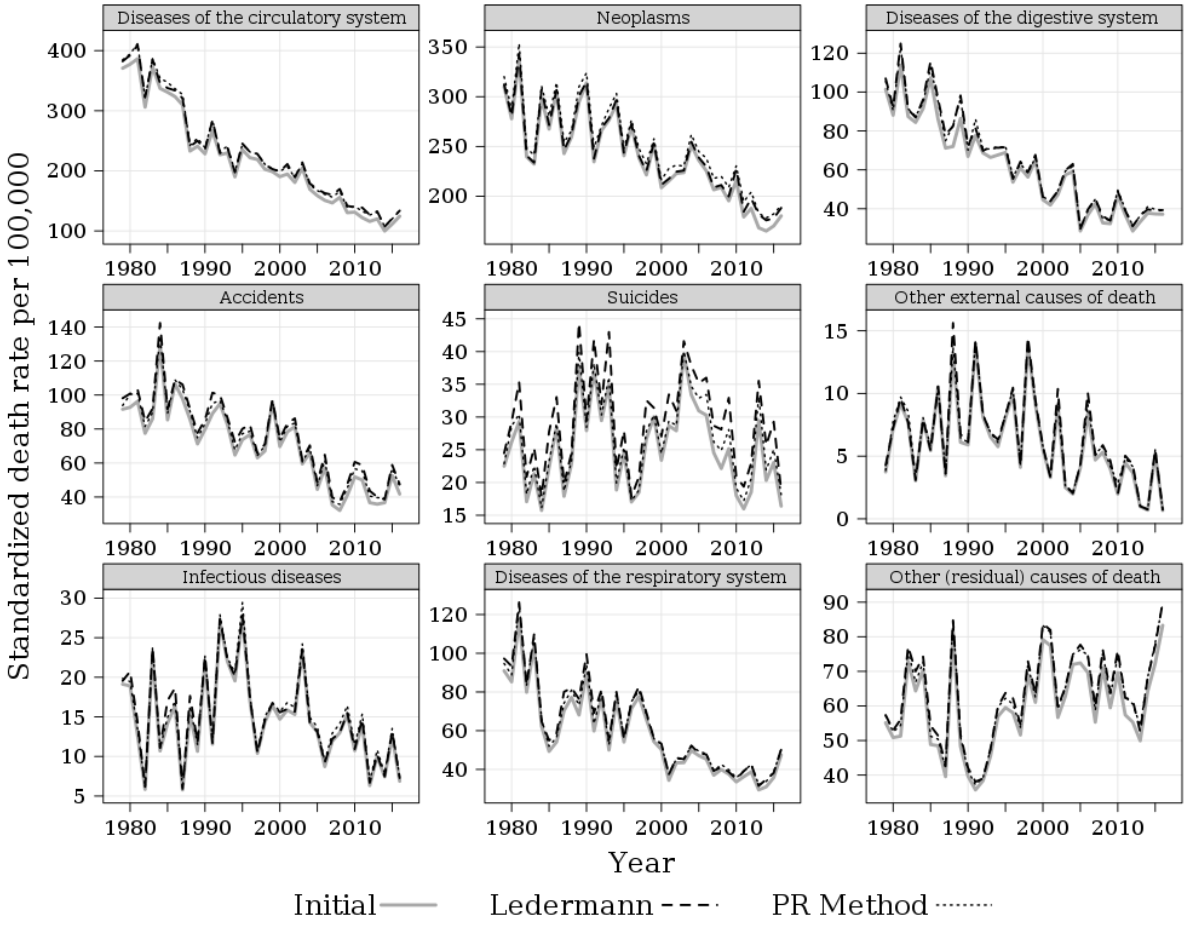

FEMALES

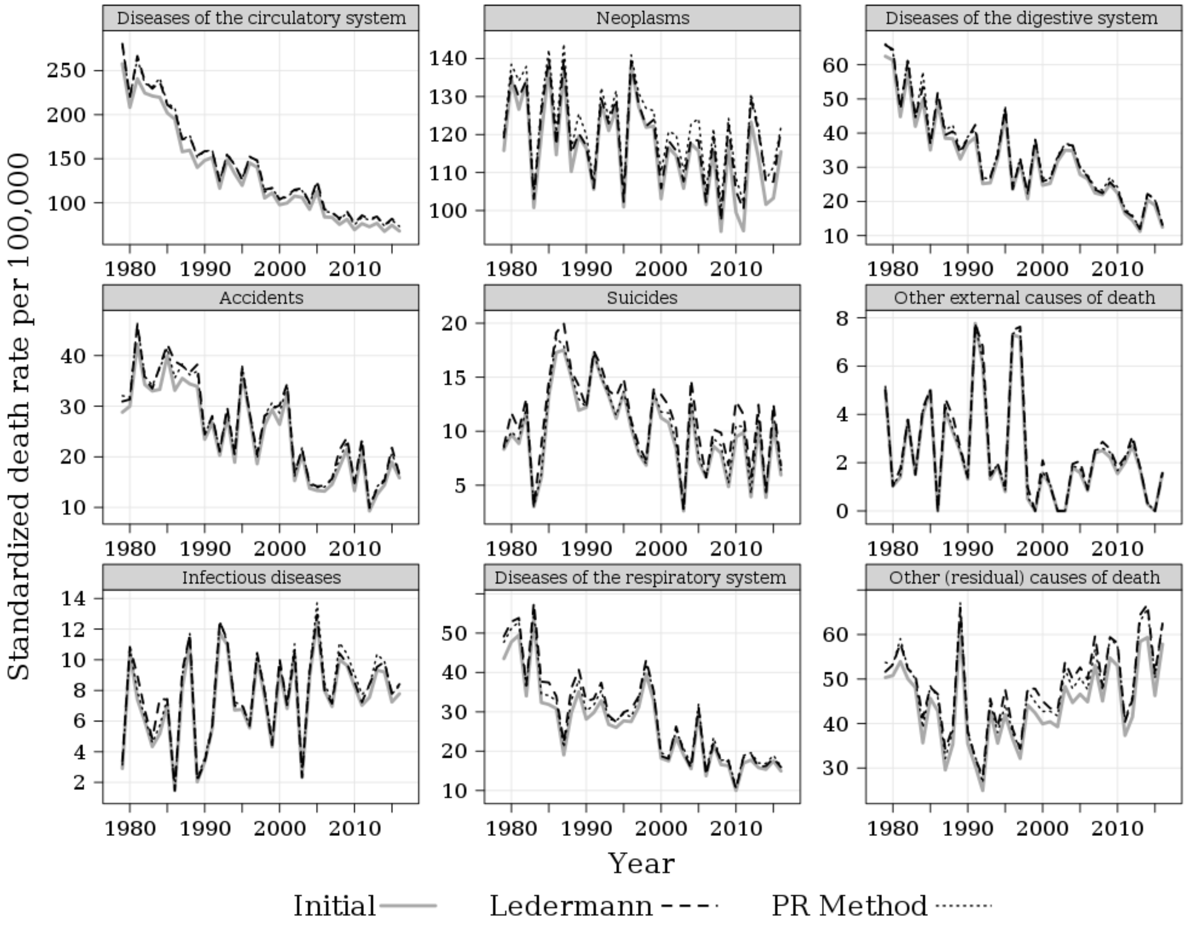

Diseases of the circulatory system
Neoplasms
Diseases of the digestive system
Accidents
Suicides
Other external causes of death
Infectious diseases
Diseases of the respiratory system
Other (residual) causes of death
Standardized death rate per 100,000
Year
Initial
Ledermann
PR Method

Lozère

MALES

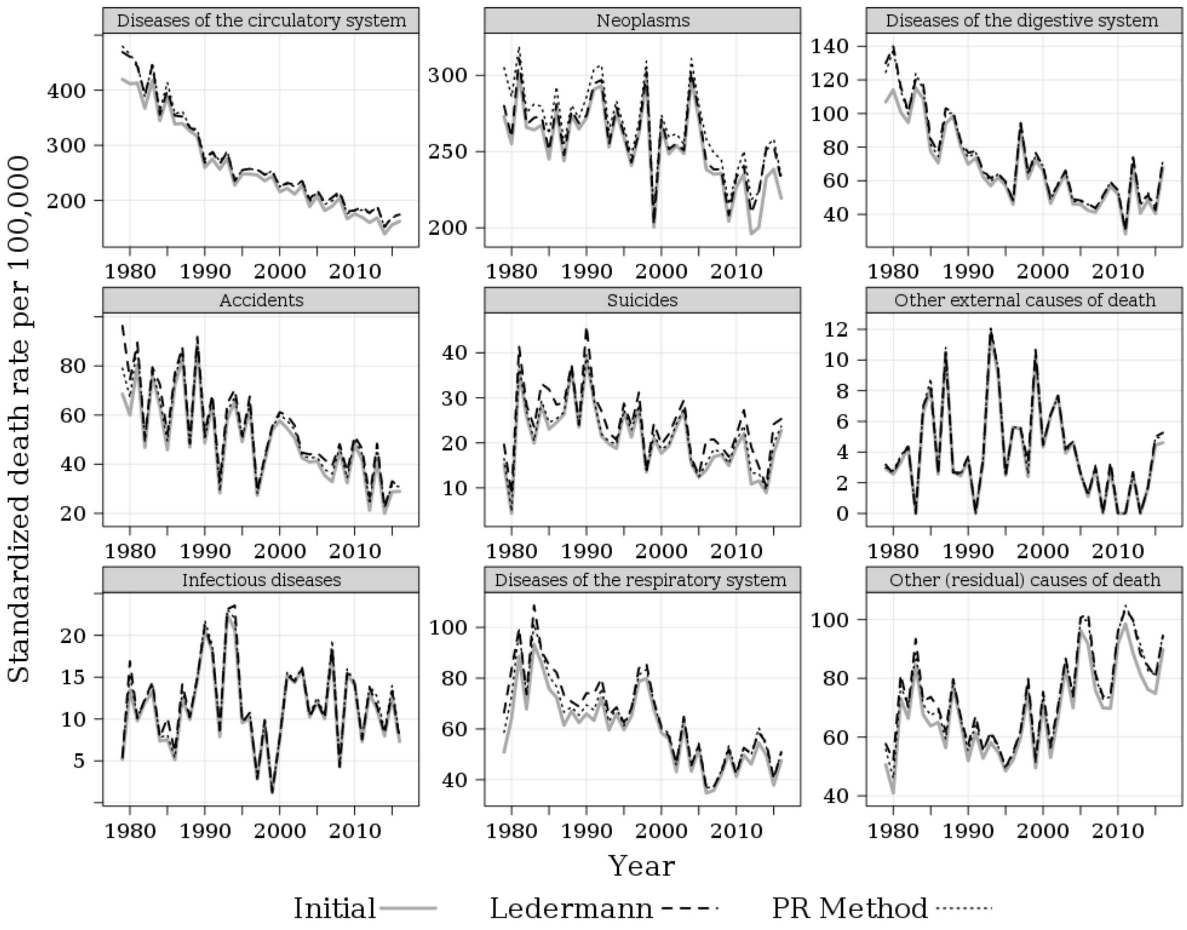

FEMALES

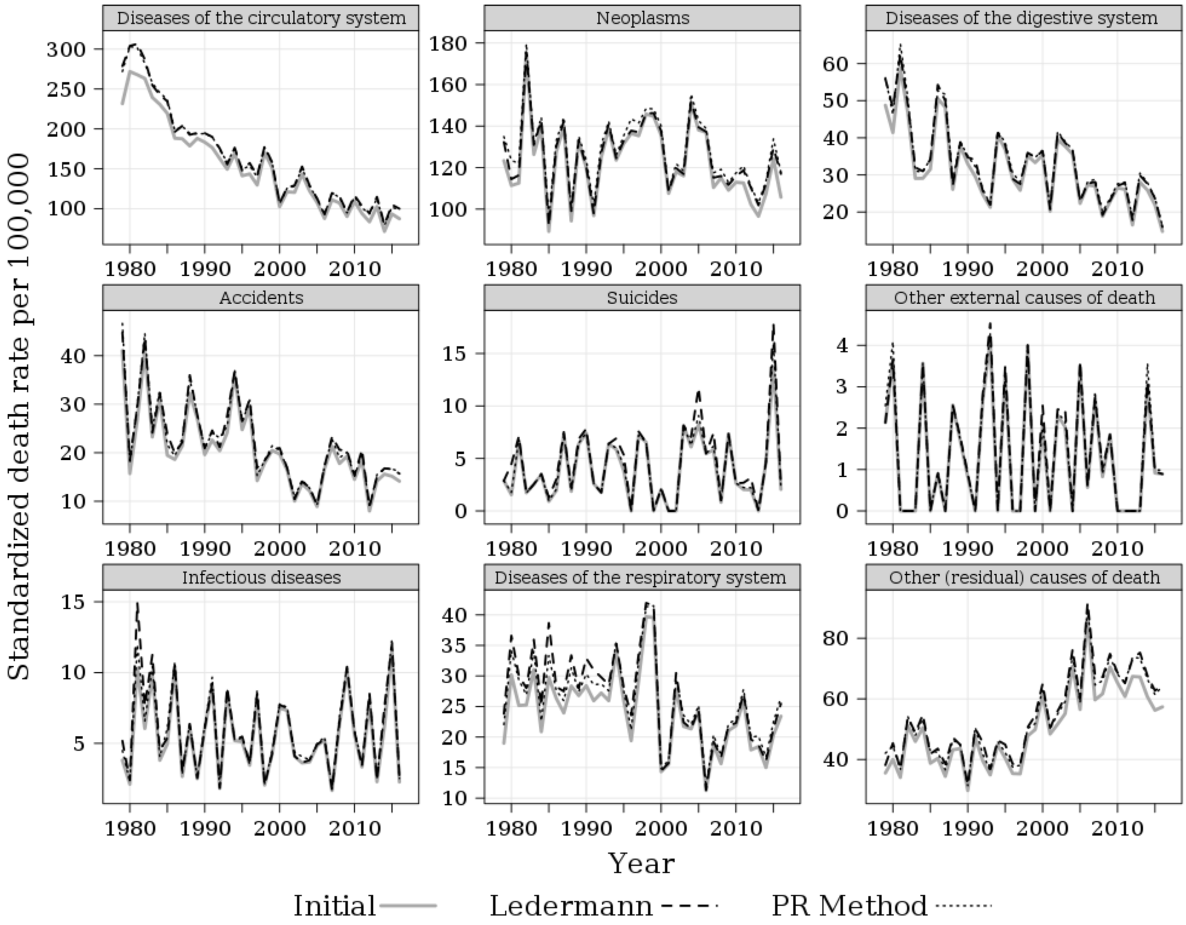

Diseases of the circulatory system
Neoplasms
Diseases of the digestive system
Accidents
Suicides
Other external causes of death
Infectious diseases
Diseases of the respiratory system
Other (residual) causes of death
Standardized death rate per 100,000
Year
Initial
Ledermann
PR Method

Paris

MALES (Figure 4 in the main text)

FEMALES

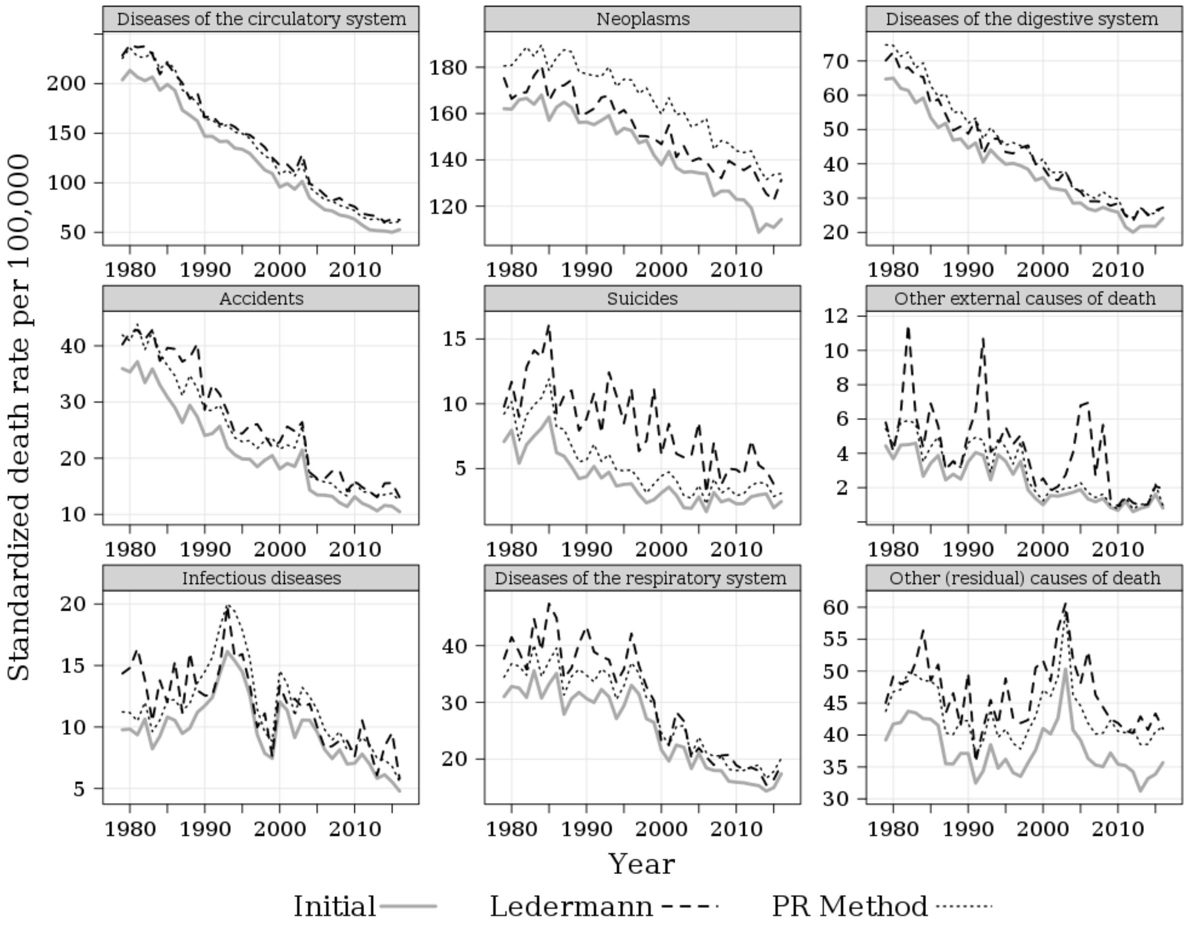

Pas-de-Calais

MALES

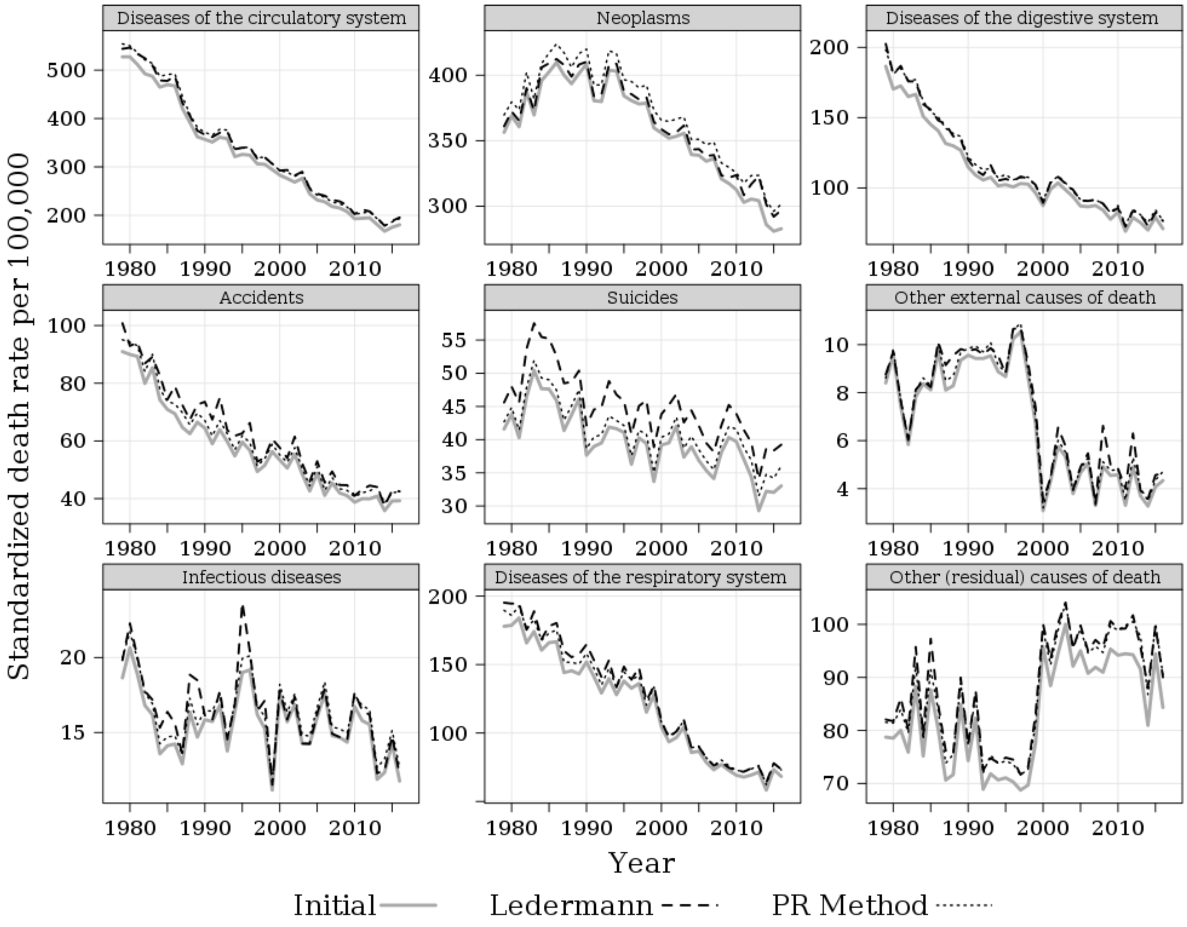

FEMALES

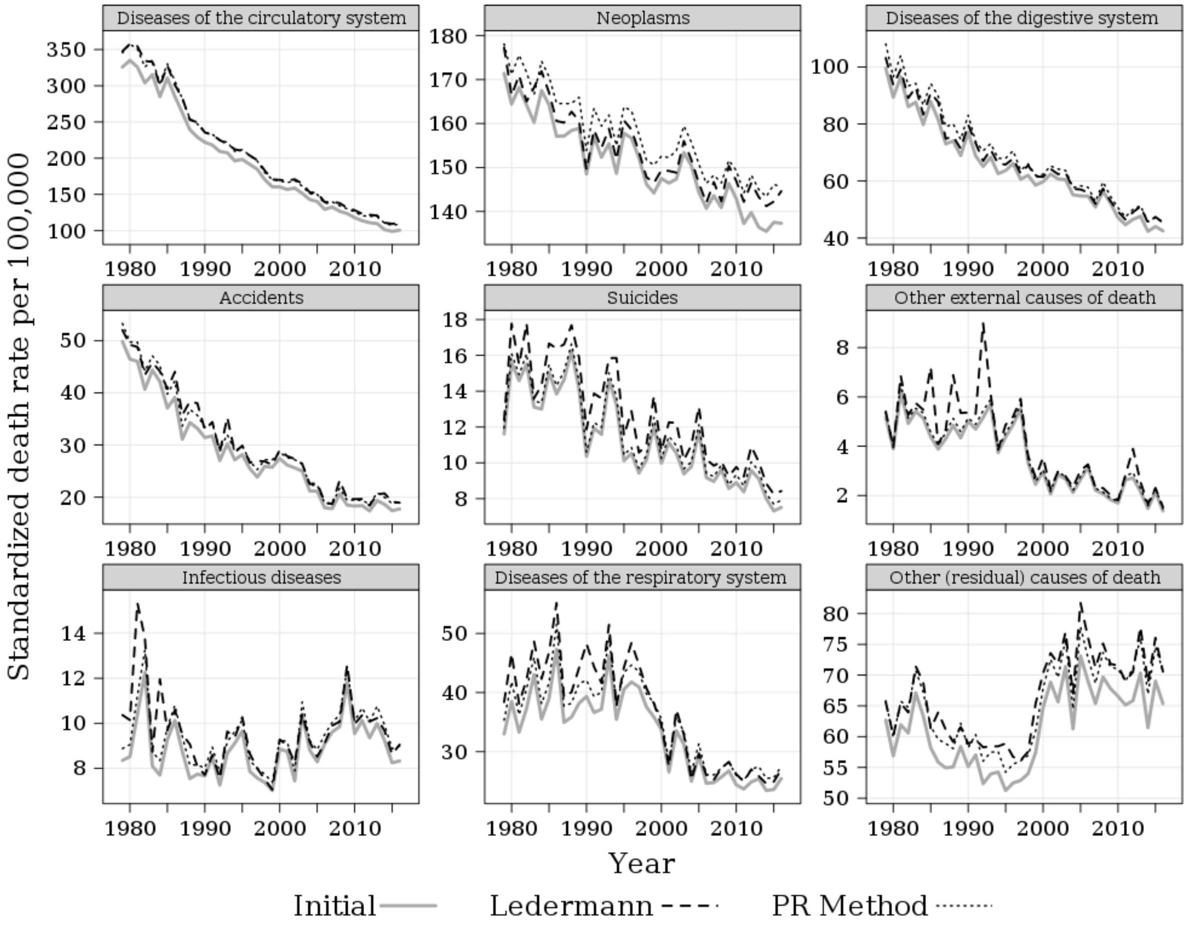
Diseases of the circulatory system
Neoplasms
Diseases of the digestive system
Accidents
Suicides
Other external causes of death
Infectious diseases
Diseases of the respiratory system
Other (residual) causes of death
Standardized death rate per 100,000
Year
1980
1990
2000
2010
Initial
Ledermann
PR Method

Seine-Saint-Denis

MALES

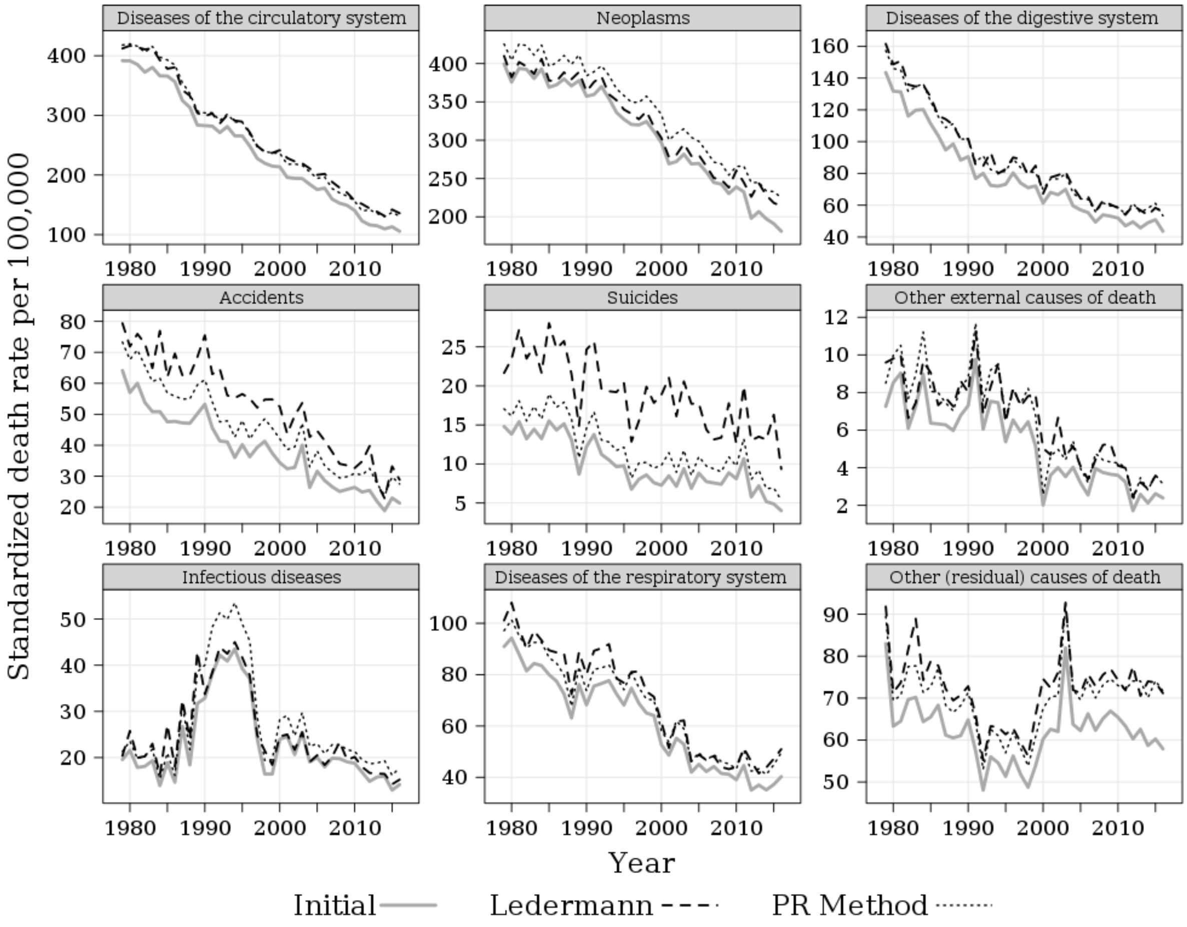

FEMALES

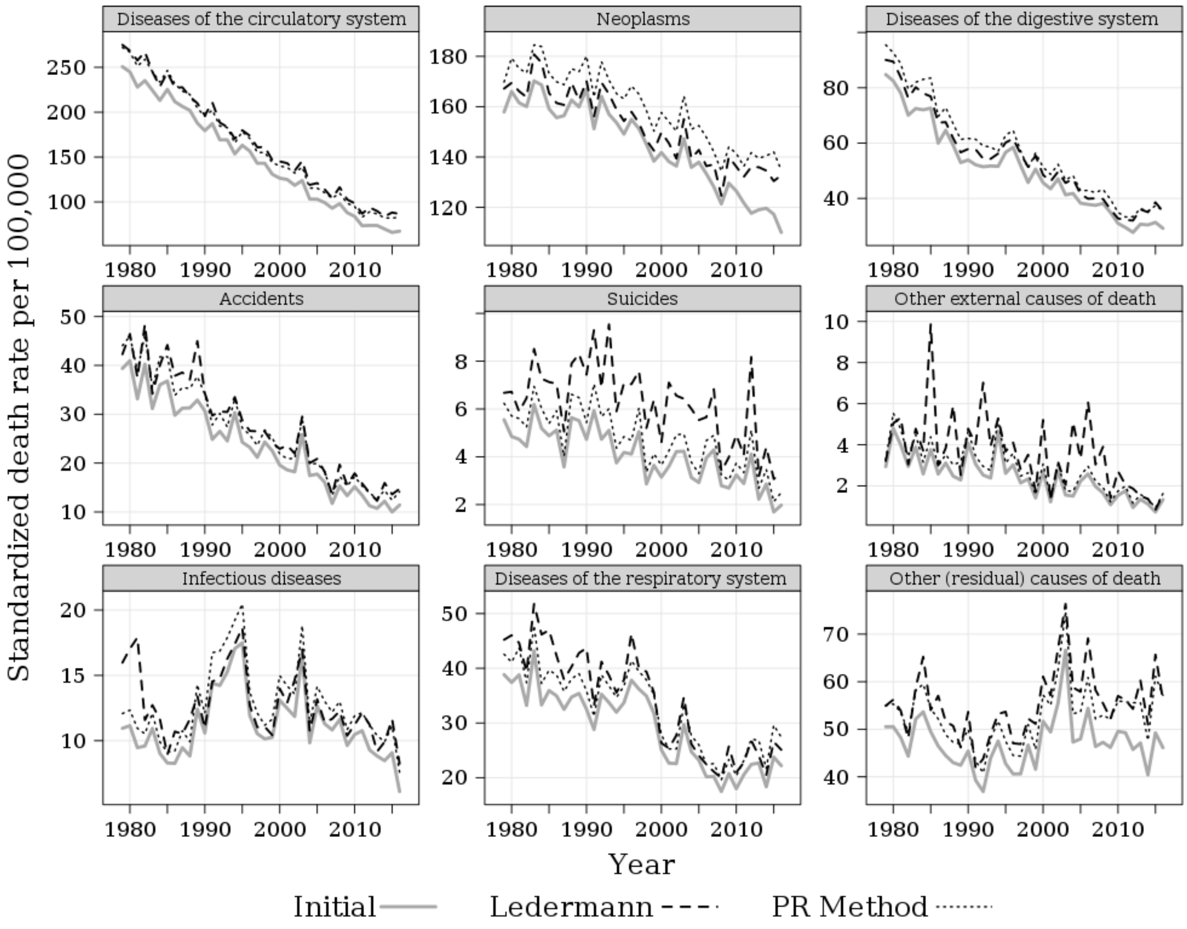

Diseases of the circulatory system
Neoplasms
Diseases of the digestive system
Accidents
Suicides
Other external causes of death
Infectious diseases
Diseases of the respiratory system
Other (residual) causes of death
Standardized death rate per 100,000
Year
Initial
Ledermann
PR Method

France

MALES (Figure 3 in the main text)

FEMALES

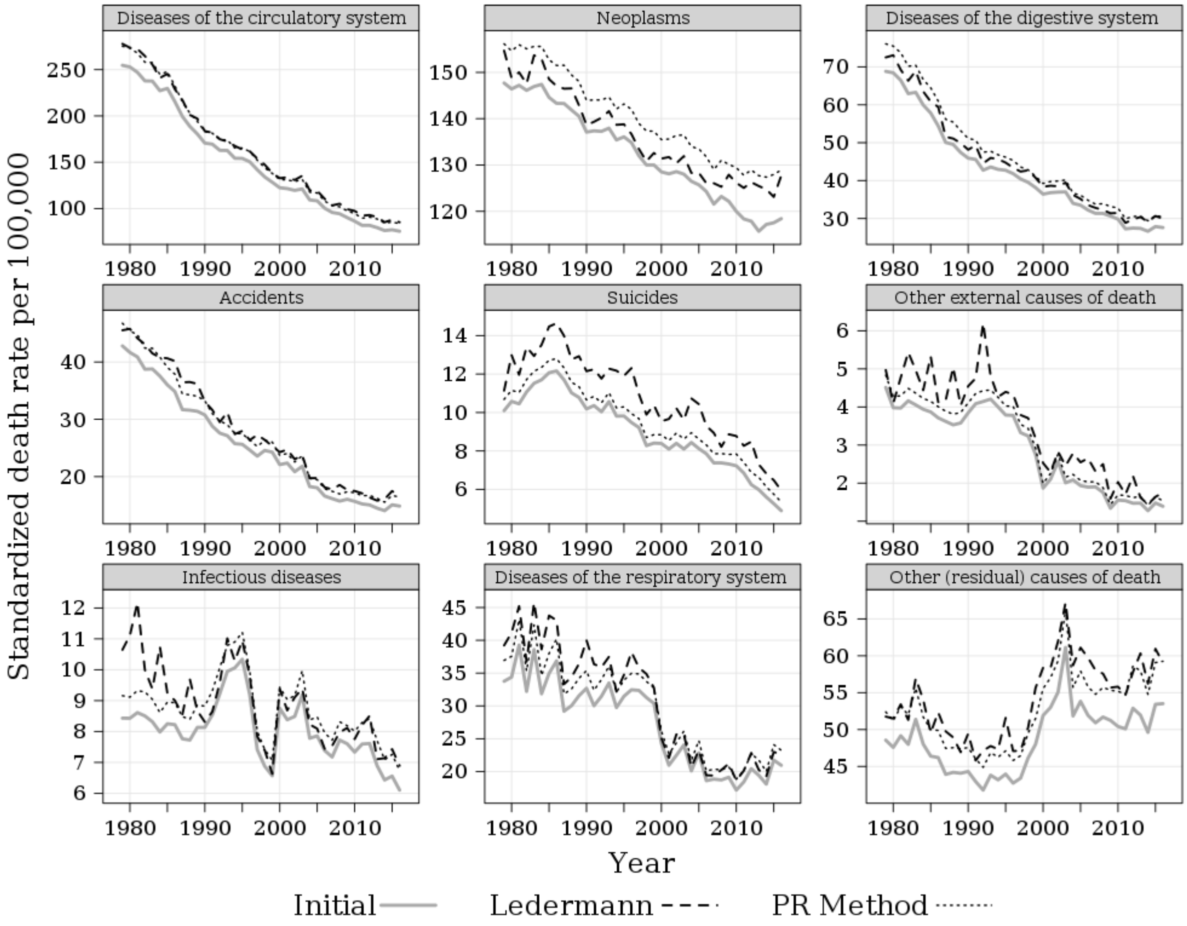

A. Results of the simulation model: performance depending on the number of regions (N)

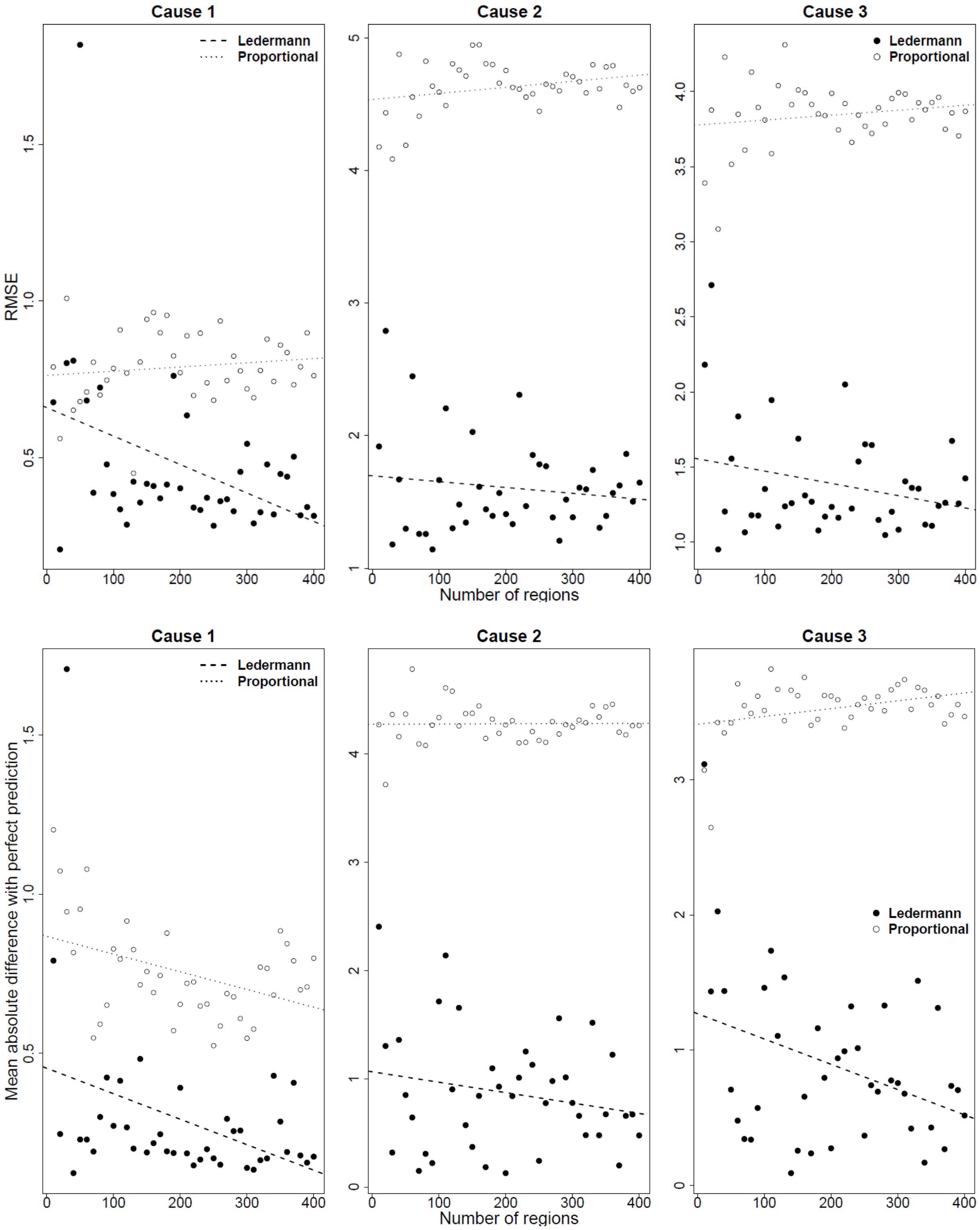

B. Results of the simulation model: performance depending on the share of ill-defined causes

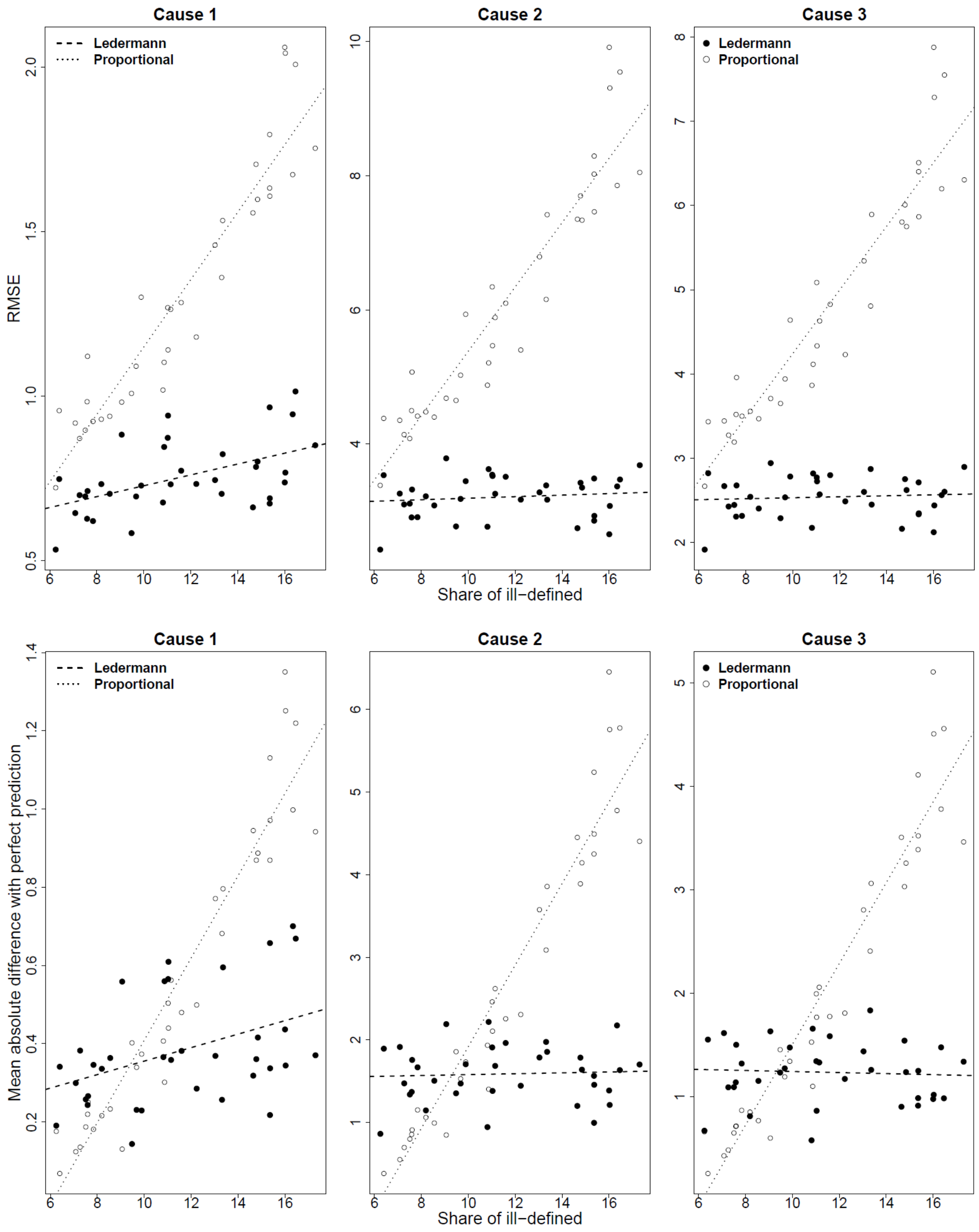

Supplement: Supplemental Material [file RPST_A_2332629_SM7071.pdf]
